# SHAPE-Seq 2.0: systematic optimization and extension of high-throughput chemical probing of RNA secondary structure with next generation sequencing

**DOI:** 10.1093/nar/gku909

**Published:** 2014-10-10

**Authors:** David Loughrey, Kyle E. Watters, Alexander H. Settle, Julius B. Lucks

**Affiliations:** School of Chemical and Biomolecular Engineering, Cornell University, Ithaca, NY 14850, USA

## Abstract

RNA structure is a primary determinant of its function, and methods that merge chemical probing with next generation sequencing have created breakthroughs in the throughput and scale of RNA structure characterization. However, little work has been done to examine the effects of library preparation and sequencing on the measured chemical probe reactivities that encode RNA structural information. Here, we present the first analysis and optimization of these effects for selective 2′-hydroxyl acylation analyzed by primer extension sequencing (SHAPE-Seq). We first optimize SHAPE-Seq, and show that it provides highly reproducible reactivity data over a wide range of RNA structural contexts with no apparent biases. As part of this optimization, we present SHAPE-Seq v2.0, a ‘universal’ method that can obtain reactivity information for every nucleotide of an RNA without having to use or introduce a specific reverse transcriptase priming site within the RNA. We show that SHAPE-Seq v2.0 is highly reproducible, with reactivity data that can be used as constraints in RNA folding algorithms to predict structures on par with those generated using data from other SHAPE methods. We anticipate SHAPE-Seq v2.0 to be broadly applicable to understanding the RNA sequence–structure relationship at the heart of some of life's most fundamental processes.

## INTRODUCTION

RNAs play diverse functional roles in many natural cellular processes (1), and are being increasingly engineered to control these processes in many synthetic biology and biotechnology applications (2). This diverse function of RNA is intimately connected to its ability to fold into intricate structures. Recently, high-throughput techniques that combine nuclease digestion (3–5) or chemical probing (6–9) with next-generation sequencing have started to shed new light on the sequence/structure relationship of RNA. Because of the inherent multiplexing and enormous throughput offered by sequencing-based approaches, these techniques are providing some of the first ‘genome-wide’ snapshots of RNA structure (9,10)—effectively bringing RNA structural biology into the ‘omics’ era (11).

**Figure 1.**
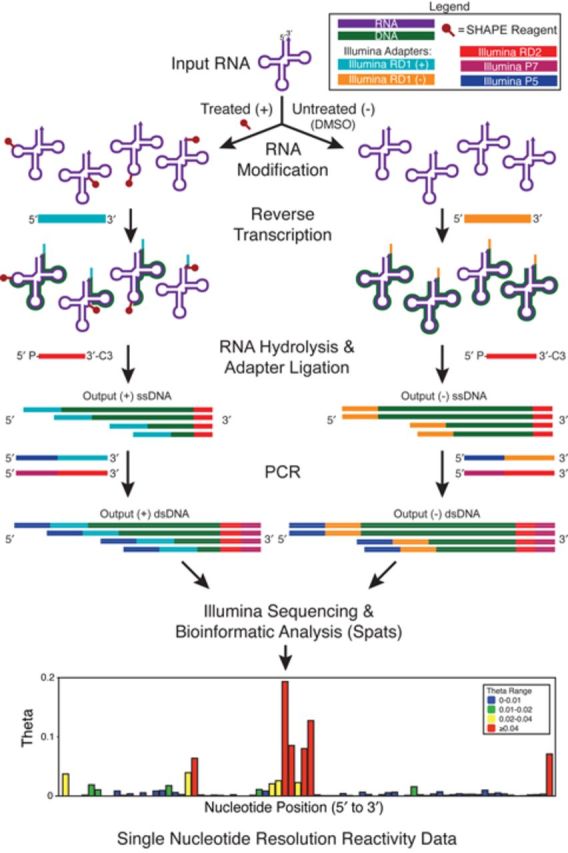
The basic SHAPE-Seq protocol. In SHAPE-Seq (6,7), RNAs are modified with chemical probes, such as 1M7 (24), or any probe that covalently modifies the RNA in a structure-dependent fashion (12). RT of the RNAs creates a pool of cDNAs, whose length distribution reflects the distribution of modification positions. Control reactions are performed to account for RT fall-off at unmodified positions. RT primer tails contain a portion of one of the required Illumina sequencing adapters, while the other is added to the 3′ end of each cDNA through a single-stranded DNA ligation. A limited number of PCR cycles are used to both amplify the library and add the rest of the required adapters prior to sequencing. A freely available bioinformatic pipeline Spats (7,26–27) is then used to align sequencing reads, correct for biases due to RT-based signal decay (26,27) and calculate reactivity spectra for each RNA. See Supplementary Figures S2–S4 for protocol details.

Of these techniques, those that favor chemical probing over nuclease digests show the most promise because of the inherent versatility (12), higher resolution and *in vivo* accessibility (9–10,13–15) of many chemical probes. Several such techniques have been developed (selective 2′-hydroxyl acylation analyzed by primer extension sequencing (SHAPE-Seq) (6,7), DMS-Seq (9–10,13), MAP-Seq (8)) that each follow the same general protocol consisting of: (i) structure-dependent modification of the RNA *in vitro* or *in vivo*; (ii) reverse transcription (RT) of the modified RNA into a cDNA pool whose length distribution reflects the location of modifications; (iii) sequencing library construction, involving the addition of platform-specific adapter sequences to the cDNA pool, optional amplification with polymerase chain reaction (PCR) and quality control assessment steps; (iv) sequencing of the library and (v) bioinformatic processing of sequencing reads and calculation of reactivity spectra for each RNA (Figure 1). While proving to be powerful, these sequencing-based techniques are complex and involve many more steps, including ligation and PCR, than approaches that use electrophoretic analysis (Supplementary Figure S1) (16–18). Very little work has been done to evaluate the impact of these extra steps.

In this work, we systematically analyze and optimize SHAPE-Seq, and present a new version, SHAPE-Seq v2.0, that can obtain reactivity information for every nucleotide of an RNA without requiring an internal RT priming site. We start by analyzing SHAPE-Seq in the context of a panel of RNAs used in previous benchmarking of chemical probing techniques (19–21). Specifically, we systematically investigate steps of the SHAPE-Seq protocol that differ from more traditional methods that could affect measured reactivity data, including sequence context effects of adapter ligation, adapter ligation conditions, RT primer modifications and PCR. During this process, we optimize several of these steps, and show that they do not appear to be a source of differences between SHAPE-Seq and electrophoresis-based SHAPE analysis. We also show that SHAPE-Seq is highly reproducible, and report replicate reactivity spectra for each RNA in the panel.

Finally, we present SHAPE-Seq v2.0 and show that it recapitulates SHAPE-Seq reactivity spectra, but without requiring a specific RT priming site to be present on the target RNAs. SHAPE-Seq v2.0 significantly expands the capability of SHAPE-Seq by allowing it to be performed through a ‘kit’-like protocol independent of the RNAs studied. We also show that SHAPE-Seq v2.0 reactivity data can be readily incorporated into RNA structure prediction algorithms to give experimentally constrained predicted folds that are highly accurate, and on par with traditional SHAPE constrained folding (20).

## MATERIALS AND METHODS

### RNA preparation

For SHAPE-Seq v1.0, RNAs were generated through *in vitro* transcription reactions with T7 RNA polymerase. DNA templates consisted of a preceding 17-nucleotide T7 promoter, an optional 14-nucleotide 5′ structure cassette sequence (22), the desired RNA coding sequence and an optional 43-nucleotide 3′ structure cassette sequence (7,22) (Supplementary Table S1). DNA templates were generated by PCR [1 ml; containing 20 mM Tris (pH 8.4), 50 mM KCl, 2.5 mM MgCl_2_, 200 μM each dNTP, 500 nM each forward and reverse primer, 5 pM template and 0.025 U/μl Taq polymerase; denaturation at 94°C, 45 s; annealing 55°C, 30 s and elongation 72°C, 1 min; 34 cycles]. The PCR product was recovered by ethanol precipitation and resuspended in 150 μl of TE [10 mM Tris (pH 8.0), 1 mM ethylenediaminetetraacetic acid (EDTA)]. Transcription reactions (1.0 ml, 37°C, 12–14 h) contained 40 mM Tris (pH 8.0), 20 mM MgCl_2_, 10 mM DTT, 2 mM spermidine, 0.01% (vol/vol) Triton X-100, 5 mM each NTP, 50 μl of PCR-generated template, 0.04 U/μl SuperaseIN RNase Inhibitor (Ambion) and 0.1 mg/ml of T7 RNA polymerase. The RNA products were purified by denaturing polyacrylamide gel electrophoresis (8% polyacrylamide, 7 M urea, 35 W, 3 h), excised from the gel using an appropriately placed sacrifice lane for ultraviolet shadowing and recovered by passive elution and ethanol precipitation. The purified RNA was resuspended in 50 μl TE, and concentrations were measured with the Qubit fluorimeter. All of the RNAs with the flanking structure cassettes contained a unique four-nucleotide bar code to multiplex SHAPE-Seq v1.0 experiments as described previously (7) (Supplementary Table S1). In general, QuSHAPE and SHAPE-Seq v1.0 experiments were performed on RNAs containing the optional structure cassettes, while SHAPE-Seq v2.0 experiments were performed on RNAs without these cassettes (Supplementary Table S1).

For SHAPE-Seq v2.0 RNAs, each RNA sequence was altered to begin with GG, and cloned between the T7 RNA promoter and Hepatitis δ (HepD) antigenomic ribozyme and PCR amplified. The resulting templates were transcribed *in vitro* using T7 RNA polymerase as above, and purified by standard gel excision methods (23). For the HepC IRES and cyclic-di-GMP Riboswitch, standard run-off transcription was performed without the ribozyme to obtain higher yields. A table of all RNA sequences used in this study can be found in Supplementary Table S1.

### RNA modification

For the initial benchmarking and optimization studies, all RNAs were folded and modified individually with 1-methyl-7-nitroisatoic anhydride (1M7) (6.5 mM, final) in batches (24). The RNAs (50 pmol in 60 μl) were denatured by heating at 95°C for 2 min, and snap-cooled on ice for 60 s before the addition of 30 μl of folding buffer. The sample was refolded in the 1× folding buffer (10 mM MgCl_2_, 100 mM NaCl and 100 mM HEPES (pH 8.0)), in a total volume of 90 μl for 20 min at 37°C. These reaction volumes were split and added to 5 μl 1M7 (10×, 65 mM in dry dimethyl sulfoxide (DMSO)) and 5 μl dry DMSO to form the (+) and (–) reactions, respectively. Reactions were complete after 70 s at 37°C. RNAs were recovered by the addition of 50 μl of water, 10 μl of 3M NaOAc, 1 μl of 20 mg/ml glycogen and 300 μl of 100% ethanol, followed by incubation at –80°C for 30 min and centrifugation (15k rpm) at 4°C for 30 min. Multiple batches of the same RNAs were combined into an overall stock of 700 pmol RNA (350 pmol unmodified (+), 350 pmol modified (–), each in 70 μl TE), and stored at –20°C until use (<3 weeks).

For v1.0 replicate experiments, RNAs were folded as described above. For v2.0, RNAs were folded in 20 pmol batches in 12 μl, with the addition of the folding buffer bringing the total volume to 18 μl. In addition, only 1 μl 1M7 and 1 μl dry DMSO were used for the (+) and (–) reactions and recovery required the addition of 90 μl H_2_O instead of 40 μl. Buffer conditions and ligand concentrations for each RNA are listed in Supplementary Table S2.

### QuSHAPE

For QuSHAPE experiments, 10 pmol from the modified (+) and unmodified (–) batches of RNA were suspended in 10 μl of water. Two sequencing lanes were also established with 10 pmol of purified RNA in 9 μl of water. RT reaction mixtures were prepared with the addition of 3 μl of 0.3 μM Vic-labeled [(+) and one of the sequencing lanes, usually ddT] or Ned-labeled [(–) and the other sequencing lane, usually ddA] RT primer, with sequence GAACCGGACCGAAGCCCG. Primers were annealed following denaturation at 95°C for 2 min and 65°C for 5 min and immediate snap-cooling. Primer extension reactions were preformed by the addition of 1 μl of Superscript III, 4 μl of 5× Superscript First Strand Buffer, 0.4 μl of dNTPs at 10 mM each (dATP, dCTP, dTTP, dITP), 1 μl of 0.1 M DTT and 0.6 μl of water. Following previous capillary electrophoresis methods, dITP was used instead of dGTP to reduce band compression and increase resolution of primer extension products by capiliary electrophoresis (25). Note that 1 μl of 10 mM pertinent di-deoxy stocks (usually ddATP or ddTTP) was added to the appropriate sequencing lanes as well. The reaction mixtures (total volume of 20 μl) were incubated at 45°C for 1 min, 52°C for 25 min and 65°C for 5 min. Then 4 μl of 50 mM EDTA (pH 8.0) was added to each sample, and oppositely labeled primers (i.e. modified and ddA; unmodified and ddT) combined, precipitated with ethanol, resuspended in 10 μl of Hi-Di formamide and resolved on an Applied Biosystems 3730xl capillary electrophoresis instrument. Raw capillary electrophoresis traces were processed using QuSHAPE software as described in Karabiber *et al.* (18). QuSHAPE reactivities were then converted to QuSHAPE *θ*'s by dividing by a normalization factor so that they summed to 1 over the range of nucleotides for which reactivity data was obtained.

### SHAPE-Seq RT

RT conditions differed based on the particular library construction strategy. The details of RT primers and adapter configurations for SHAPE-Seq library preparation strategies can be found in Supplementary Figures S2–S4. For SHAPE-Seq v1.0 (Supplementary Figures S2 and S3), the general procedure for RT was carried out following the primer extension protocol in Mortimer *et al.* (7). The total amount of primer used in primer extension reactions was 9 pmol (3 μl of 3 μM primer), with the RNA concentration being 50 pmol in 10 μl. Primers were annealed by incubation at 95°C for 2 min and at 65°C for 5 min. Primer extension reactions were performed by the addition of 200 U of Superscript III, 4 μl of 5× Superscript First Strand Buffer, 0.4 μl of dNTPs at 10 mM each (dATP, dCTP, dTTP, dGTP), 1 μl of 0.1 M DTT and 0.6 μl of water. The reaction mixtures (total volume of 20 μl) were incubated at 45°C for 1 min, 52°C for 25 min and 65°C for 5 min. After primer extension, RNA was hydrolyzed by adding NaOH (1 μl, 4 M) and incubating for 5 min at 95°C. cDNAs were ethanol precipitated and resuspended in 71 μl of nuclease-free water.

For SHAPE-Seq v2.0 (Supplementary Figure S4), RNAs were not purified after the modification step. Instead, a linker sequence was added to the 3′ end of the RNA template via 5′-App Ligation by adding 6.5 μl 50% PEG 8000, 2 μl 10× T4 RNA Ligase Buffer (NEB), 1 μM 5′ App IDT miRNA linker 2 (5′App-CACTCGGGCACCAAGGAC-3′ddC) and 0.5 μl T4 RNA Ligase, truncated KQ (NEB) directly to the modified RNA. Samples were incubated overnight at room temperature, recovered by ethanol precipitation and resuspended in 10 μl of nuclease-free water. RT was then carried out as above using 1.5 pmol RT primer (3 μl at 0.5 μM) complementary to the linker sequence, and containing flanking Illumina adapter sequence and custom internal barcodes to create unique 3′ end alignments and increase randomness during Illumina sequencing (Supplementary Figure S4 and Table S3). Hydrolysis was performed the same way, but partially quenched with 1.5 μl of 1 M HCl. After EtOH precipitation, the cDNAs were dissolved in 22.5 μl nuclease-free water.

### SHAPE-Seq second adapter ligation

Adapter ligations differed based on the particular library construction strategy (Supplementary Figures S2–S4). In all SHAPE-Seq v1.0 cases, adapter sequences were ligated to each cDNA using a ssDNA ligase (CircLigase, Epicentre Biotechnologies) [100 μl; 50 mM MOPS (pH 7.5), 10 mM KCl, 5 mM MgCl_2_, 1 mM DTT, 0.05 mM ATP, 2.5 mM MnCl_2_, 5 μM adapter and 200 U ligase] and incubating for 6 h at 68°C in a thermal cycler. For SHAPE-Seq v2.0, the ligation was performed in 30 μl by mixing 50 mM MOPS (pH 7.5), 10 mM KCl, 5 mM MgCl_2_, 1 mM DTT, 0.05 mM ATP, 2.5 mM MnCl_2_, 1.67 μM adapter (Supplementary Figure S4) and 100 U ligase, incubated at 60°C for 2 h. Separate ligation reactions were carried out for the (+) and (−) cDNA library pools. The ligation reactions were stopped by heating to 80°C for 10 min, recovered by ethanol precipitation and resuspended in 20 μl of nuclease-free water. Excess adapter was removed using Agencourt Ampure XP beads following the manufacturer's protocol, eluting with 20 μl TE.

### SHAPE-Seq PCR, library QC and sequencing

Ligated libraries were then used as inputs into PCR reactions using 6, 9, 12 or 20 cycles of PCR amplification as indicated in Results. PCR primers contained sequences required for Illumina sequencing and index multiplexing as indicated in Supplementary Figures S2–S4. A 50 μl PCR reaction contained 2.5 μl of cDNA template, 1 μl of 100 μM forward and reverse primers, 1 μl of 10 mM dNTPs, 10 μl 5× Phusion Buffer, 33.5 μl water and 1 U Phusion DNA polymerase (NEB). Multiple reactions were made together and split before the pertinent number of PCR amplification cycles. PCR reactions were cleaned up with Agencourt Ampure XP beads following the manufacturer's protocol, eluting with 20 μl TE. No direct size selection was performed on the resulting adapter-ligated library. Libraries were assayed for quality in one of two methods: (i) using an Agilent Bioanalyzer 2100 high-sensitivity DNA chip to compare 9- and 12-cycle amplification, looking for characteristic peaks and peak enrichment as described in Mortimer *et al.* (7) or (ii) using fluorescently labeled PCR primers to generate fragments to be analyzed by capillary electrophoresis, looking for the same features.

The 9-cycle PCR amplification products (unless otherwise indicated in Results) were then sequenced on an Illumina MiSeq or HiSeq platform following the manufacturer's standard cluster generation and sequencing protocols. For runs that consisted of multiple RNAs, (+) and (–) channels were balanced for molarity, and loaded to reach a final concentration of at least 2 ng/μl. This was then diluted according to the standard Illumina sequencing protocols.

### SHAPE-Seq data analysis

Fastq files generated from the Illumina sequencing process were analyzed using the freely available software package Spats (spats.sourceforge.net), as described in (7,26–27). Spats takes paired-end fastq files and performs bioinformatics read alignment and a maximum-likelihood-based signal decay correction (6–7,26–27) to calculate SHAPE-Seq *θ* values for each nucleotide of an RNA. In SHAPE-Seq experiments, reactivity data are presented as $\Theta = \{ \theta _i ;i = 1..L\}$, which is a probability distribution over the length of an RNA, *L*, representing the probability, *θ_i_*, that each nucleotide, *i*, is modified by the modification reagent (26,27). SHAPE-Seq *θ*'s are similar to more traditional SHAPE reactivity data (typically referred to as a set of reactivity numbers for each nucleotide, $\{ r_i \}$), except that *θ*'s are constrained to sum to 1 over the length of the RNA since they represent a probability distribution, i.e. $\sum\nolimits_{i = 1}^L {\theta _i = 1}$. Thus, *θ*'s are independent of scale factors typically used to define SHAPE reactivities (19,21,28–29), and can be rigorously calculated from the observed (+) and (–) fragment distributions in a SHAPE-Seq experiment (7,26–27).

For this work, we used Spats v0.8.0, which contains an updated adapter trimming algorithm. Once installed, a typical spats command was executed by entering:python adapter_trimmer.py –A-b-sequence <second_adapter_sequence> –A-t-sequence <first_adapter_sequence> –read-len 35 R1.fastq R2.fastq RNA_targets.faspats –num-mismatches 0 -o Output RNA_targets.fa RRRY YYYR combined_R1.fastq combined_R2.fastq where <second_adapter_sequence> is the sequence of the second adapter, <first_adapter_sequence> is the sequence of the first adapter, R1.fastq and R2.fastq are the fastq sequencing files and RNA_targets.fa is the FASTA formatted file containing the RNA sequences under study. Spats outputs text files containing (+) and (–) fragment counts and *θ*’s, for each position in each RNA in the targets file. Fragment distributions were calculated by dividing the fragment counts at each position by the total number of fragments observed in the channel, so that the fragment distribution summed to 1 over the length of the RNA.

### Computational modeling

The *Fold* executable of the RNAStructure (30) software package (version 5.5) was used to calculate SHAPE-Seq-constrained RNA secondary structures. For each RNA, SHAPE-Seq v2.0 *θ*'s were first converted into *ρ* values by multiplying by the length of the RNA, *L* (Equation (1)). Due to the inability to uniquely align reads containing a single nucleotide, *L* was one nucleotide less than the full length of the RNA used in experiments. The flags ‘-sh’, ‘-sm’ and ‘-si’ were used to input the SHAPE-Seq *ρ* data file, slope *m* and intercept *b*, respectively. The parameters *m* and *b* were used to define the pseudo-free energy $\Delta G_i = m\ln (\rho _i + 1) + b$ that was used in the minimum free energy (MFE) structure calculation (20,28–30). The sensitivity and positive predictive value (PPV) of each predicted RNA structure was evaluated using the RNAStructure (30) *Scorer* executable (version 5.5), using the established crystallographic secondary structure as the accepted structure (19–21). The entire RNA sequence was used in these calculations except for nucleotides at the very 5′ end of the RNAs that were included for RNA synthesis using T7 polymerase. A table of RNA sequences used can be found in Supplementary Table S1.

## RESULTS

### Investigation and optimization of SHAPE-Seq library preparation

There are two distinct protocol steps associated with sequencing library preparation that distinguish SHAPE-Seq from SHAPE analyzed by capillary electrophoresis (Figure 1, Supplementary Figure S1): adapter ligation and PCR. Here, we sought to investigate if these steps impact the measured fragment distributions and reactivities for a set of RNAs that have been used in other recent SHAPE benchmarking experiments (6–7,19–21). The starting panel, chosen for the abundance of crystallographic and SHAPE structural data available in the literature, consisted of the RNAse P specificity domain from *Bacillus subtilis*, unmodified tRNA^phe^ from *Escherichia coli*, the P4-P6 domain of the *Tetrahymena* group I intron ribozyme, the 5S rRNA from *E. coli* and the aptamer domain from the *Vibrio vulnificus* adenine riboswitch (Supplementary Table S1).

#### Adapter ligation

One of the most distinct differences between SHAPE-Seq and capillary electrophoresis methods like QuSHAPE (18) is the ligation of a second adapter sequence required for Illumina sequencing (Figure 1, Supplementary Figure S1). The previously published SHAPE-Seq protocol (SHAPE-Seq v1.0) uses single-stranded DNA (ssDNA) ligation to add a 61 nt ssDNA oligonucleotide to the 3′ end of the cDNA required for sequencing. This oligo consists of the Illumina RD2 and P7 sequences used for priming sequencing and flow cell binding, respectively (Supplementary Figure S2) (7). It was hypothesized that this ligation step could introduce bias in SHAPE-Seq measurements due to sequence or structure-specific ligation efficiencies.

We began by comparing SHAPE-Seq v1.0 reactivity spectra to QuSHAPE reactivities for each panel RNA, using the same starting pools of modified (+) and unmodified (–) RNAs. Overall there was a strong degree of correlation between the two methods for each of the RNAs and we did not find any systematic differences in reactivity spectra that would suggest ligation was causing biases (Supplementary Figure S5).

**Figure 2.**
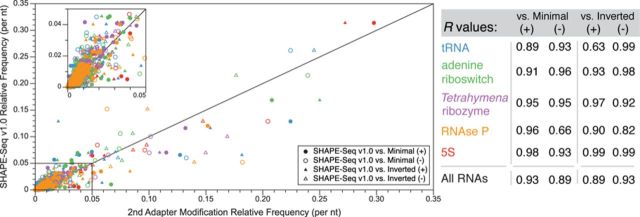
A comparison of SHAPE-Seq v1.0 to adapter ligation variations. SHAPE-Seq libraries on the same pools of modified and unmodified RNAs were constructed and sequenced using the standard adapter configuration (v1.0), a shortened second adapter (Minimal) or an adapter layout where sequences were switched between RT primer tail and second ligation (Inverted) (Supplementary Figure S2). For each RNA and each configuration, the (+) and (–) fragment distributions (per nucleotide frequencies) are compared. Circles represent the v1.0 versus Minimal comparison and triangles represent the v1.0 versus Inverted comparison. Filled/open symbols are for (+)/(–) fragment distributions, respectively, and are color coded according to the RNA as indicated in the table, which contains Pearson's correlation (*R*) values for the comparisons.

We therefore tested two other adapter variants: a truncated ‘Minimal’ adapter (25 nt) (Supplementary Figure S2b) and an ‘Inverted’ layout where the RT primer tail and the adapter sequences were switched (Supplementary Figure S2c). These libraries tested whether the adapter length (Minimal), or sequence (Inverted) would contribute to potential ligation bias, while staying within the sequence constraints of the Illumina platform. After constructing and sequencing these libraries from the same modified and unmodified RNA pools, we compared cDNA fragment length distributions, since changes in ligation conditions only affect the sampling of the modified or unmodified RNA fragments (Figure 2). The aggregate Pearson's correlation coefficients (*R*) across all RNAs in the panel show that these distributions are in good agreement, with *R* values ranging from 0.89 to 0.93. Comparisons between adapter variants for individual RNAs also showed a strong correlation, with values ranging from 0.82 to 0.99, except for two specific cases: RNase P v1.0 versus Minimal (–) (*R* = 0.66) and tRNA v1.0 versus Inverted (+) (*R* = 0.63) (Figure 2). These discrepancies for the RNAse P case mostly arose from differences between the ends of the (–) distribution, though these differences do not cause major discrepancies in the overall {*θ_i_*} calculation (*R* = 0.92) (Supplementary Figure S6a). Like RNAse P, the tRNA distributions only show major differences at the 5′ and 3′ ends, but these differences do cause a discrepancy in the {*θ_i_*} calculation (*R* = 0.68) (Supplementary Figure S6b).

While constructing these libraries, we also investigated the ligation conditions in general, including enzyme choice, ligation temperature and specific blocking groups used to prevent adapter concatenation during ligation. Previous work by Kwok *et al.* demonstrated that there was much room for improvement in the ssDNA ligation step, and devised an alternate T4 DNA ligase-based method (31). However, our concern that secondary structures could potentially cause uncharacterized ligation bias at the low temperatures required for T4 DNA ligase (31) led us to instead improve the CircLigase reaction originally used in SHAPE-Seq v1.0 (7). We began by performing a time course assay on the SHAPE-Seq v1.0 ligation reaction. We ligated the 61 nt SHAPE-Seq v1.0 Illumina adapter (Supplementary Figure S2) to a 126 nt cDNA for varying amounts of time (Supplementary Figure S7) and found a saturation in ligated product after 1–2 h. We next compared CircLigase II to CircLigase I for potential improved ligation efficiency (Supplementary Figure S8). A 2 h ligation of an RT primer to the Illumina 61 nt adapter at 68°C was compared to ligation at 60°C for each ligase. Both ligases performed better at 60°C, with Circligase I at 60°C being the optimal condition tested (Supplementary Figure S8).

We also investigated blocking groups on the 5′ end of the RT primer, and 3′ end of the Illumina adapter (Supplementary Figure S2) for their importance in preventing concatemer formation during ligation (Supplementary Figures S9 and S10). First, an unblocked or biotin-blocked RT primer was ligated to the 25 nt Minimal Illumina adapter (Supplementary Figure S9). Though the unblocked and blocked RT primers performed equivalently, we proceeded with the biotin-blocked primer (Supplementary Figure S9). We next tested 3′ di-deoxy-cytosine, phosphate and 3-carbon spacer modifications to the Illumina second adapter designed to prevent adapter concatemerization (Supplementary Figure S10). We ligated the blocked adapter to an RT primer that was either 5′ blocked with biotin or unblocked, using CircLigase I. All 3′ modifications showed some degree of concatemer formation, with the phosphate modification producing the most concatemer (Supplementary Figure S10). We recommend the 3-carbon spacer modification, as it displayed lower amounts of concatemer formation and is less expensive than di-deoxy-cytosine.

#### PCR amplification

Another distinct difference between SHAPE-Seq and SHAPE analyzed by capillary electrophoresis is the use of PCR to build and amplify SHAPE-Seq libraries before sequencing. PCR can be a powerful feature of SHAPE-Seq library construction, as it can amplify low signals (32), add custom barcodes or library indexes or complete adapter sequences as in the Minimal library above (Supplementary Figure S2c). However, PCR could introduce a systematic bias in the SHAPE-Seq measurement since certain fragments can be preferentially amplified.

**Figure 3.**
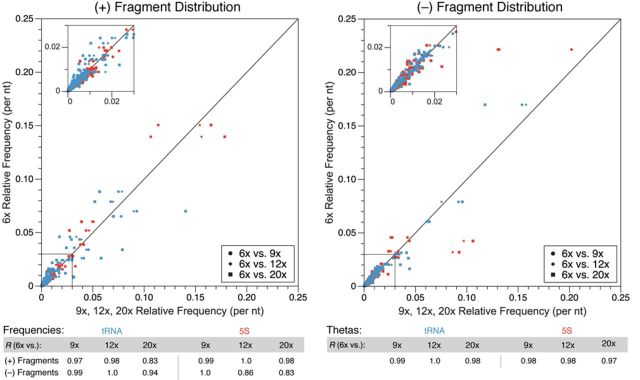
Characterization of varying numbers of PCR cycles in SHAPE-Seq library construction. SHAPE-Seq v1.0 libraries were constructed for tRNA and 5S rRNA using either 6×, 9×, 12× or 20× cycles of PCR before sequencing. (+) and (–) fragment distributions for each RNA were compared between 6× cycles and the other cycle numbers as in Figure 2. Pearson's correlation values (*R*) for individual comparisons between fragment distributions, and {*θ_i_*} distributions, are shown on the bottom.

To test the effects of PCR on measured fragment distributions, we created SHAPE-Seq v1.0 libraries for tRNA and 5S rRNA using either 6, 9, 12 or 20 cycles of PCR amplification before sequencing (Figure 3). A comparison between the 6× and 9× (+) and (–) distributions for each RNA showed near perfect agreement, with *R* values ranging from 0.97 to 1.0 (Figure 3), indicating that the PCR cycles used in SHAPE-Seq v1.0 (9×) are not biasing these distributions. The comparison between 6× and 12× was similar, with the *R* values ranging from 0.86 to 1.0. Even the comparison between 6× and 20× showed strong agreement, with *R* values ranging from 0.83 to 0.98. For all of the worst comparisons, with *R* values of 0.83 to 0.86, the discrepancy stemmed mostly from a few positions rather than a systematic length bias across the lengths of the RNAs. Specifically, the shortest fragments for the 5S 12× (–) and 20× (–) fragment distributions differed slightly (Supplementary Figure S11). For the tRNA (+) distribution, there were six positions that showed a similar discrepancy (Supplementary Figure S11). Regardless of these discrepancies in the (+) and (–) distributions, the comparisons between {*θ_i_*} values between the different PCR cycle conditions yielded *R* values in the range of 0.97–1.0 (Figure 3), indicating that up to 20 PCR cycles can be used without the introduction of systematic bias in SHAPE-Seq reactivities.

### Assessing the reproducibility of SHAPE-Seq

Replicate indexed SHAPE-Seq experiments were performed using a similar configuration as the minimal adapter library above, except that a 34 nt adapter sequence was used instead to take advantage of Illumina TruSeq multiplexing (Supplementary Figure S3). Replicates, measured from completely independent library preparations, were obtained for the five panel RNAs discussed above, as well as four additional RNAs—the cyclic di-GMP bacterial riboswitch from *V. cholerae*, the TPP riboswitch from *E. coli*, the SAM I riboswitch from *T. tencongensis* and the Hepatitis C virus IRES domain—all of which have been used in previous SHAPE benchmarking experiments (19–21). As indicated in Experimental Procedures, folding conditions included ligands where appropriate (Supplementary Table S2). The results showed that the SHAPE-Seq technique is highly reproducible for all nine RNAs (Figure 5, Supplementary Figure S12).

**Figure 4.**
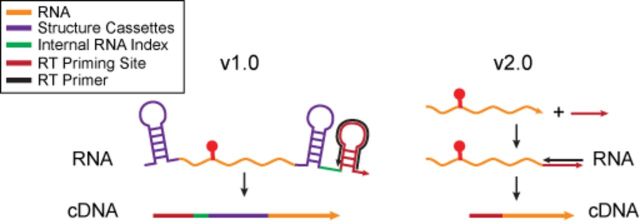
Schematic of traditional SHAPE/SHAPE-Seq v1.0 RT priming strategies and the universal RT priming strategy of SHAPE-Seq v2.0. Traditional strategies use sequences that are part of the RNA, or added structure cassette flanking sequences to prime RT reactions. In SHAPE-Seq v2.0, a linker sequence is added to the RNA post-modification, which serves as a priming site for the RT reaction (see Supplementary Figure S4).

**Figure 5.**
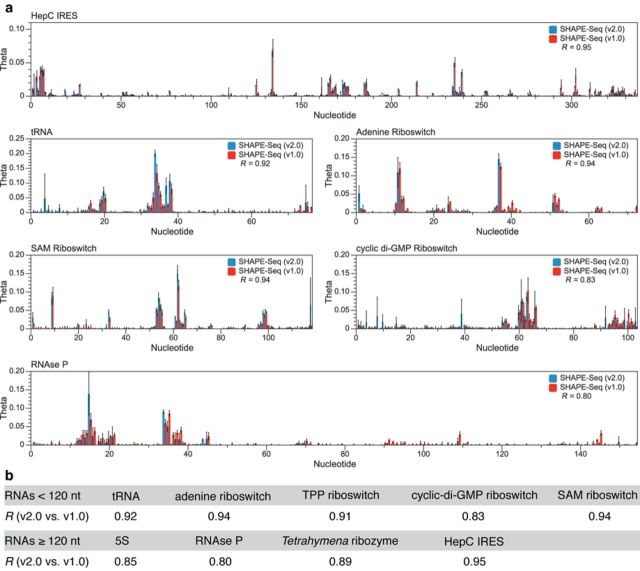
SHAPE-Seq v2.0 versus SHAPE-Seq v1.0. (a) Reactivity spectra *θ*'s are plotted for each RNA, including error bars which are calculated as standard deviations of reactivities at each nucleotide from three independent replicate experiments for SHAPE-Seq v2.0 (blue) and SHAPE-Seq v1.0 (red). Pearson's correlations from the comparisons of average reactivity spectra are shown in each plot and listed in the table in (b). Supplementary Figure S12 shows detailed comparisons for each RNA in the panel.

### SHAPE-Seq v2.0: removing RNA sequence requirements with universal RT priming

One major limitation to the SHAPE methods described above is the requirement of priming the RT step either within the RNA sequence itself, or by including structure cassette sequences that contain a 3′ RT primer binding site (Figure 4) (22). In the case of priming within the RNA itself, custom primers must be used for each RNA, and structural information is lost at the site of RT priming. In the structure cassette case, flanking sequences must be added to the RNA itself, with the potential to alter the folded structures of the RNAs. SHAPE-Seq v2.0 creates a ‘universal’ RT primer binding site by ligating a linker sequence to the 3′ end of the RNA after modification (Figure 4, Supplementary Figure S4).

#### 3′ ligation methods and other protocol adjustments

We focused specifically on ligating pre-adenylated linkers using truncated T4 RNA ligase 2 to prevent unwanted side reaction products. We tested ligation conditions using three previously designed and tested miRNA cloning linkers (33,34) available from Integrated DNA Technologies. As shown in Supplementary Figure S13, each IDT linker was effective at ligating to the tRNA^phe^ sequence. We chose linker 2 for having the highest melting temperature with respect to its complementary RT primer.

The addition of the 3′ ligation step required a number of protocol adjustments to improve cDNA yield and reduce side products. A critical adjustment was reducing the amount of RT primer used (see Experimental Procedures). This had the effect of reducing the amount of unextended primer left over after RT, and thus reducing the amount of unwanted side product, which increases the usable signal from the sequencing run. As described above in Experimental Procedures, we also altered many other intermediate steps of SHAPE-Seq, culminating in SHAPE-Seq v2.0 (Supplementary Figure S4).

#### Comparison of SHAPE-Seq v2.0 and v1.0

Reactivity spectra generated with SHAPE-Seq v2.0 and v1.0 are compared in Figure 5. Pearson's correlation coefficients were between 0.80 and 0.95, supporting a strong correlation between v1.0 and v2.0 results. Of the RNAs with weaker correlations (namely, cyclic-di-GMP Riboswitch (*R* = 0.83), RNAse P (*R* = 0.80) and 5S rRNA (*R* = 0.85)), the main qualitative differences tend to be at the ends of the RNA molecule, with v2.0 tending toward higher *θ* values at the 5′ end, and v1.0 higher at the 3′ end (Figure 5, Supplementary Figure S12).

### Using SHAPE-Seq reactivities as constraints in thermodynamic RNA folding algorithms

While our primary goal was to assess the ability of SHAPE-Seq to generate accurate and reproducible reactivity data in a high-throughput manner, it is important to recognize downstream uses of this information. One common use is to constrain thermodynamic RNA folding algorithms (19–21,28–29,35). In this approach, each SHAPE reactivity, *r_i_*, of an RNA is converted into a $\Delta G_{SHAPE,i} = m{\rm ln}(r_i + 1) + b$, which are then used to predict RNA secondary structural properties, such as the MFE structure. The parameters *m* and *b* are fit to produce the most accurate structural predictions over a benchmark set of RNAs for which reactivity information is available (28). Secondary structure prediction accuracy is assessed by comparing the predicted MFE structure to the crystal structure using two representative statistical measures: sensitivity, or the fraction of base pairs in the accepted (crystal) structure predicted correctly; and PPV, which is the fraction of predicted pairs that are correct (35). Overall, the incorporation of SHAPE reactivity data into thermodynamic structure prediction algorithms has been shown to increase the accuracy of predictions (19–21,28–29).

To incorporate SHAPE-Seq reactivity data into folding algorithms, we converted *θ*'s to a scale that is more similar to the reactivity scale typically used for SHAPE experiments. In traditional SHAPE data scaling, ‘highly reactive’ positions are set to a reactivity of ∼1 by scaling to a normalization factor that averages the reactivities of these positions while excluding outliers (29). In SHAPE-Seq, *θ*'s are guaranteed to be <1 due to the constraint that they sum to 1 over the length of the RNA, thus SHAPE-Seq *θ*'s are smaller than typical reactivities. However, *θ*'s can be easily converted to a similar scale by multiplying *θ*'s by the length of the RNA, *L*. Defining

(1) \begin{equation*} \rho _i = L\theta _i \end{equation*}

ensures that the average *ρ_i_*, $\bar \rho$, is

(2) \begin{equation*} \bar \rho = \frac{{\sum\nolimits_{i = 1}^L {\rho _i } }}{L} = L\frac{{\sum\nolimits_{i = 1}^L {\theta _i } }}{L} = 1 \end{equation*}

making *ρ*'s on roughly the same scale as SHAPE *r*’s. Therefore, we used SHAPE-Seq v2.0 *ρ*'s to evaluate sensitivity and PPV predictions for each RNA in the panel (Table 1). As seen from Table 1, when we used the current recommended values of *m* = 1.8 and *b* = −0.6, we observe an increase in the total sensitivity and PPV values over unconstrained folds. These values are in fact comparable to results from recent studies that used QuSHAPE reactivity with these parameters (20). However, since *ρ*'s are slightly different than SHAPE *r*’s, we anticipated that there could be room to adjust *m* and *b* values. We found that *m* = 1.1 and *b* = −0.3 gave total sensitivity and PPV values over the panel to be 84% and 89%, respectively, with many RNAs in the panel predicted to a very high sensitivity and PPV individually (Table 1, Supplementary Table S6). We emphasize that *m* = 1.1 and *b* = –0.3 should serve as a *guide* for incorporating SHAPE-Seq reactivity data into folding algorithms, though more work should be performed to refine these values over a broader context of RNA structures.

**Table 1.** RNA structure prediction accuracy using the RNAStructure (30) *Fold* algorithm and the SHAPE-Seq v2.0 reactivity data (*ρ*’s) as constraints, with different *m* and *b* parameters

RNA	Total sensitivity	Total PPV
No SHAPE data	228/360 = 63.3%	229/373 = 61.4%
SHAPE parameters (*m* = 1.8 and *b* = −0.6)	292/360 = 81.1%	293/351 = 83.5%
SHAPE-Seq updated parameters (*m* = 1.1 and *b* = −0.3)	303/360 = 84.2%	304/342 = 88.9%

Sensitivity and PPV values for each RNA are in Supplementary Tables S4–S6.

## DISCUSSION

In this work, we present a systematic analysis and optimization of the SHAPE-Seq technique to structurally characterize RNAs in a high-throughput, multiplexed fashion. Overall, the above results demonstrate that our optimizations to the previously published SHAPE-Seq v1.0 technique (6,7) provide highly reproducible nucleotide resolution chemical reactivity data over the wide array of structural contexts present in our panel of benchmark RNAs (Figure 5).

Initial comparisons between reactivities generated from before SHAPE-Seq v1.0 optimization and QuSHAPE showed there was a strong overall correlation between the two methods, with specific differences highlighted by the inspection of individual reactivity spectra. As shown in Supplementary Figure S5, for most of the RNAs, SHAPE-Seq v1.0 and QuSHAPE capture the same clusters of reactive nucleotides, but differ in the specific *θ* value assigned to these positions. There does, however, appear to be a difference between the two techniques at low reactivity nucleotides. In particular, there are a large number of nucleotide positions (216 out of a total of 586) where the SHAPE-Seq *θ* is 0, while the QuSHAPE *θ* is a small, non-zero number. We hypothesize that the analog nature of the capillary electrophoresis read-out in QuSHAPE experiments, and the Gaussian fitting algorithm used to quantify electopherogram peaks, can amplify baseline noise and make zero reactivity peaks appear to have a small but non-zero reactivity.

We systematically optimized each step of the SHAPE-Seq v1.0 protocol associated with library preparation and sequencing, which constitute the major differences between SHAPE-Seq and SHAPE analyzed by capillary electrophoresis. As shown above, steps such as adapter ligation (Figure 2) and PCR (Figure 3) do not appear to introduce a systematic bias in SHAPE-Seq reactivity data.

We also sequenced three SHAPE-Seq v2.0 libraries on both the HiSeq and MiSeq platforms. All three show a very strong correlation between the HiSeq and MiSeq for the (+) and (–) read distributions, with *R* values for these comparisons all in excess of 0.99 (Supplementary Figure S14). This indicates that the choice of sequencing platform has no effect on SHAPE-Seq data.

We then expanded the flexibility of the SHAPE-Seq technique by incorporating the standard Illumina library indexing strategy to sequence multiple libraries in the same lane (Supplementary Figures S3 and S4). This can be used in a number of ways, for example, by using indexes to perform replicate experiments on the same RNA, or to run different groups of RNAs together as done in this work. In principle, this flexibility allows any number of experimental variations to be performed.

We have also significantly extended SHAPE-Seq by creating a universal RT priming strategy that does not require RNA-specific primers to be designed and used, or flanking sequence to be added to the RNA itself. This new technique, SHAPE-Seq v2.0, works by ligating a linker sequence *after* modification, which can then serve as an RT priming site. With this innovation, SHAPE-Seq v2.0 can now be performed on unknown RNAs, without the need to deal with the complexities and biases associated with random RT primers (9,10). In addition, SHAPE-Seq v2.0 allows reactivity information to be characterized for almost the entire length of the RNA, without losing structural information at RT priming sites within the RNA sequence. Finally, SHAPE-Seq v2.0 should be equally applicable to naturally synthesized RNAs as it is to *in vitro* transcribed RNAs, and thus should allow the standardization of chemical probing experimental protocols and data analysis.

The data presented in this work also represents a high-quality benchmark SHAPE-Seq data set for a panel of RNAs that are becoming the gold standard for technique comparison in the field (19–21). As described in Supplementary Table S7, all data are freely available in the RNA Mapping Database (36). This should serve as a useful resource for further experimental technique development, as well as to researchers interested in using SHAPE-Seq data to constrain computational RNA folding algorithms to give more accurate RNA structural models. In fact, as we have shown above SHAPE-Seq *ρ* values can be used to make structural predictions that are as accurate as those from more traditional SHAPE experiments (Table 1). It is our hope that this data set serves as a starting point for understanding how best to incorporate SHAPE-Seq data into computational structure prediction.

Finally, we note that while this technique was originally named ‘SHAPE-Seq’ after the SHAPE chemistry that was used in the first version of the technique, it is in fact applicable to any RNA structure-dependent chemical probe. With our innovation of the SHAPE-Seq v2.0 universal priming technique, and the already rigorous signal decay correction and accurate reactivity calculation offered by the Spats pipeline (7,26–27), we anticipate SHAPE-Seq to be continued to be used in a wide array of powerful techniques aimed at understanding the RNA sequence–structure relationship at the heart of some of life's most fundamental processes.

## SUPPLEMENTARY DATA

Supplementary Data are available at NAR Online.

SUPPLEMENTARY DATA
