# Supplementary material for: SHAPE-Seq 2.0: systematic optimization and extension of high-throughput chemical probing of RNA secondary structure with next generation sequencing
Source: Nucleic Acids Res. 2014 Oct 10;42(21):e165. doi: 10.1093/nar/gku909 (PMC4245970; doi:10.1093/nar/gku909)
Supplement: SUPPLEMENTARY DATA [file supp_42_21_e165__index.html]

SHAPE-Seq 2.0: systematic optimization and extension of high-throughput chemical probing of RNA secondary structure with next generation sequencing — SHAPE-Seq 2.0: systematic optimization and extension of high-throughput chemical probing of RNA secondary structure with next generation sequencing — SUPPLEMENTARY DATA 

# SHAPE-Seq 2.0: systematic optimization and extension of high-throughput chemical probing of RNA secondary structure with next generation sequencing

## SUPPLEMENTARY DATA

**Files in this Data Supplement:**

- SUPPLEMENTARY DATA
