# Supplementary material for: SHAPE-Seq 2.0: systematic optimization and extension of high-throughput chemical probing of RNA secondary structure with next generation sequencing
Source: Nucleic Acids Res. 2014 Oct 10;42(21):e165. doi: 10.1093/nar/gku909 (PMC4245970; doi:10.1093/nar/gku909)
Supplement: SUPPLEMENTARY DATA [file supp_gku909_nar-01967-met-g-2014-File007.pdf]

Supporting Information for

SHAPE-Seq 2.0: Systematic Optimization and Extension of High-Throughput Chemical Probing of RNA Secondary Structure with Next Generation Sequencing.

David Loughrey[1], Kyle E. Watters[1], Alexander H. Settle[1] and Julius B. Lucks[1]*

1 – School of Chemical and Biomolecular Engineering, Cornell University, Ithaca NY 14850

Table/Figure	Description
Table S1	RNA sequences used in this study.
Table S2	RNA folding buffer conditions and ligand concentrations used in replicate experiments.
Figure S1	SHAPE-CE Flowchart
Figure S2	SHAPE-Seq v1.0 and Second Adapter Variation Library (Minimal, Inverted) Construction Schematics.
Figure S3	SHAPE-Seq v1.0 library indexing strategy.
Figure S4	SHAPE-Seq v2.0 Library Construction Schematic.
Table S3	List of barcoded reverse transcription primers used during the SHAPE-Seq v2.0 library generation.
Figure S5	QuSHAPE vs. SHAPE-Seq (v1.0) detailed comparisons.
Figure S6	SHAPE-Seq v1.0 vs. Minimal or Inverted adapter variations for RNase P and tRNA.
Figure S7	Time course of CircLigase I ligation efficiency.
Figure S8	Ligase comparison for addition of SHAPE-Seq second adapter.
Figure S9	Modification and optimization of RT primer blocking groups for adapter ligation.
Figure S10	Effect of 3' blocking group on second adapter concatemerization and ligation efficiency.
Figure S11	SHAPE-Seq v1.0 fragment distributions for different numbers of PCR cycles.
Figure S12	SHAPE-Seq v2.0 vs. SHAPE-Seq v1.0.
Figure S13	Choice of 5' adenylated linker sequence for SHAPE-Seq 2.0 universal priming strategy.
Figure S14	SHAPE-Seq v2.0 reactivities generated from the MiSeq and HiSeq platforms.
Table S4	RNA structure prediction accuracy using the RNAStructure *fold* algorithm with no SHAPE constraints
Table S5	RNA structure prediction accuracy using SHAPE-Seq v2.0 reactivity data (ρ's) as constraints in the RNAStructure *fold* algorithm, m = 1.8, b = -0.6)
Table S6	RNA structure prediction accuracy using SHAPE-Seq v2.0 reactivity data (ρ's) as constraints in the RNAStructure *fold* algorithm, m = 1.1, b = -0.3)
Table S7	Data deposition table.

Table S1: RNA sequences used in this study. Sequences are listed as DNA sequence for convenient use in Spats. Blue lettering indicates the structure cassette sequence initially developed for SHAPE analysis using capillary electrophoresis (1), or the GG sequence required for T7 RNA polymerase *in vitro* transcription. Green lettering indicates an RNA-specific barcode used for multiplexing SHAPE-Seq v1.0 experiments (2, 3). Red lettering indicates reverse transcriptase (RT) priming site. Note that the SHAPE-Seq v2.0 uses an RT priming site that is introduced after linker ligation (Figure S4), so no explicit RT priming site is present in the RNA, although it is appended to the RNA sequence before Spats analysis.

Name	Sequence	Experiments	Figure
5S rRNA, *E. coli*	GGCCTTCGGGCCAAATGCCTGGCGGCC GTAGCGCGGTGGTCCCACCTGACCCCA TGCCGAACTCAGAAGTGAAACGCCGTA GCGCCGATGGTAGTGTGGGGTCTCCCC ATGCGAGAGTAGGGAACTGCCAGGCAT CCGATCCGCTTCGGCGGATCCAAATAAA TCGGGCTTCGGTCCGGTTC	QuSHAPE, SHAPE-Seq v1.0	Figure 2, Figure 3, Figure 5, SI Figure 5, SI Figure 11, SI Figure 12, SI Figure 14
Adenine riboswitch, *V. vulnificus*	GGCCTTCGGGCCAAACGCTTCATATAAT CCTAATGATATGGTTTGGGAGTTTCTAC CAAGAGCCTTAAACTCTTGATTATGAAGT GCCGATCCGCTTCGGCGGATCCAAACA AATCGGGCTTCGGTCCGGTTC	QuSHAPE, SHAPE-Seq v1.0	Figure 2, Figure 5, SI Figure 5, SI Figure 12
Cyclic di-GMP riboswitch, *V. cholerae*	GGTGTCACGCACAGGGCAAACCATTCG AAAGAGTGGGACGCAAAGCCTCCGGCC TAAACCAGAAGACATGGTAGGTAGCGG GGTTACCGATGGCAAAATGCATACCCGA TCCGCTTCGGCGGATCCAAATCGGGCTT CGGTCCGGTTC	QuSHAPE, SHAPE-Seq v1.0	Figure 5, SI Figure 12
P4-P6, *Tetrahymena* group I intron ribozyme	GGCCTTCGGGCCAAGAATTGCGGGAAA GGGGTCAACAGCCGTTCAGTACCAAGT CTCAGGGGAAACTTTGAGATGGCCTTGC AAAGGGTATGGTAATAAGCTGACGGACA TGGTCCTAACCACGCAGCCAAGTCCTAA GTCAACAGATCTTCTGTTGATATGGATG CAGTTCAAAACCCCGATCCGCTTCGGCG GATCCAATAAAATCGGGCTTCGGTCCGG TTC	QuSHAPE, SHAPE-Seq v1.0	Figure 2, Figure 5, SI Figure 5, SI Figure 12, SI Figure 14
RNAse P, specificity domain, *B. subtilis*	GGTCGTGCCTAGCGAAGTCATAAGCTAG GGCAGTCTTTAGAGGCTGACGGCAGGA AAAAAGCCTACGTCTTCGGATATGGCTG AGTATCCTTGAAAGTGCCACAGTGACGA AGTCTCACTAGAAATGGTGAGAGTGGAA CGCGGTAAACCCCTCGACCGATCCGCT TCGGCGGATCCCTTGAAATCGGGCTTC GGTCCGGTTC	QuSHAPE, SHAPE-Seq v1.0	Figure 2, Figure 5 SI Figure 5, SI Figure 6, SI Figure 12
$tRNA^{phe}$, *E. coli*	GGCCTTCGGGCCAAGCGGATTTAGCTC AGTTGGGAGAGCGCCAGACTGAAGATC TGGAGGTCCTGTGTTCGATCCACAGAAT TCGCACCACCGATCCGCTTCGGCGGAT CCAAAGAAATCGGGCTTCGGTCCGGTTC	QuSHAPE, SHAPE-Seq v1.0	Figure 2, Figure 3, Figure 5, SI Figure 5 SI Figure 6, SI Figure 11, SI Figure 12, SI Figure 14
Hepatitis C virus IRES domain	GGCCTTCGGGCCAACCATGAATCACTCC CCTGTGAGGAACTACTGTCTTCACGCAG AAAGCGTCTAGCCATGGCGTTAGTATGA	QuSHAPE, SHAPE-Seq v1.0	Figure 5, SI Figure 12

	GTGTCGTGCAGCCTCCAGGACCCCCCC TCCCGGGAGAGCCATAGTGGTCTGCGG AACCGGTGAGTACACCGGAATTGCCAG GACGACCGGGTCCTTTCTTGGATTAACC CGCTCAATGCCTGGAGATTTGGGCGTG CCCCCGCGAGACTGCTAGCCGAGTAGT GTTGGGTCGCGAAAGGCCTTGTGGTAC TGCCTGATAGGGTGCTTGCGAGTGCCC CGGGAGGTCTCGTAGACCGTGCATCAT GAGCACGAATCCTAAACCTCAACCGATC CGCTTCGGCGGATCCAAGCAAATCGGG CTTCGGTCCGGTTC		
SAM I riboswitch, *T. tencongensis*	GGCCTTCGGGCCAATTCTTATCAAGAGA AGCAGAGGGACTGGCCCGACGAAGCTT CAGCAACCGGTGTAATGGCGATCAGCC ATGACCAAGGTGCTAAATCCAGCAAGCT CGAACAGCTTGGAAGATAAGAACCGATC CGCTTCGGCGGATCCAACCAAATCGGG CTTCGGTCCGGTTC	QuSHAPE, SHAPE-Seq v1.0	Figure 5, SI Figure 12
TPP riboswitch, *E. coli*	GGCCTTCGGGCCAAGACTCGGGGTGCC CTTCTGCGTGAAGGCTGAGAAATACCCG TATCACCTGATCTGGATAATGCCAGCGT AGGGAAGTTCCCGATCCGCTTCGGCGG ATCCATAAAAATCGGGCTTCGGTCCGGT TC	QuSHAPE, SHAPE-Seq v1.0	Figure 5, SI Figure 12
5S rRNA, *E. coli*	GGATGCCTGGCGGCCGTAGCGCGGTG GTCCCACCTGACCCCATGCCGAACTCA GAAGTGAAACGCCGTAGCGCCGATGGT AGTGTGGGGTCTCCCCATGCGAGAGTA GGGAACTGCCAGGCAT	SHAPE-Seq v2.0	Figure 5, SI Figure 12
Adenine Riboswitch, *V. vulnificus*	GGACGCTTCATATAATCCTAATGATATG GTTTGGGAGTTTCTACCAAGAGCCTTAA ACTCTTGATTATGAAGTG	SHAPE-Seq v2.0	Figure 5, SI Figure 12
Cyclic di-GMP riboswitch, *V. cholerae*	GGTGTCACGCACAGGGCAAACCATTCG AAAGAGTGGGACGCAAAGCCTCCGGCC TAAACCAGAAGACATGGTAGGTAGCGG GGTTACCGATGGCAAAATGCATAC	SHAPE-Seq v2.0	Figure 5, SI Figure 12
P4-P6, *Tetrahymena* group I intron ribozyme	GGAATTGCGGGAAAGGGGTCAACAGCC GTTCAGTACCAAGTCTCAGGGGAAACTT TGAGATGGCCTTGCAAAGGGTATGGTAA TAAGCTGACGGACATGGTCCTAACCACG CAGCCAAGTCCTAAGTCAACAGATCTTC TGTTGATATGGATGCAGTTCAAAACC	SHAPE-Seq v2.0	Figure 5, SI Figure 12
RNAse P, specificity domain, *B. subtilis*	GGTCGTGCCTAGCGAAGTCATAAGCTAG GGCAGTCTTTAGAGGCTGACGGCAGGA AAAAAGCCTACGTCTTCGGATATGGCTG AGTATCCTTGAAAGTGCCACAGTGACGA AGTCTCACTAGAAATGGTGAGAGTGGAA CGCGGTAAACCCCTCGA	SHAPE-Seq v2.0	Figure 5, SI Figure 12
$tRNA^{phe}$, *E. coli*	GGCGGATTTAGCTCAGTTGGGAGAGCG CCAGACTGAAGATCTGGAGGTCCTGTGT TCGATCCACAGAATTCGCACCA	SHAPE-Seq v2.0	Figure 5, SI Figure 12
Hepatitis C virus IRES domain	GGCCATGAATCACTCCCCTGTGAGGAAC TACTGTCTTCACGCAGAAAGCGTCTAGC CATGGCGTTAGTATGAGTGTCGTGCAGC	SHAPE-Seq v2.0	Figure 5, SI Figure 12

	CTCCAGGACCCCCCCTCCCGGGAGAGC CATAGTGGTCTGCGGAACCGGTGAGTA CACCGGAATTGCCAGGACGACCGGGTC CTTTCTTGGATTAACCCGCTCAATGCCT GGAGATTTGGGCGTGCCCCCGCGAGAC TGCTAGCCGAGTAGTGTTGGGTCGCGA AAGGCCTTGTGGTACTGCCTGATAGGGT GCTTGCGAGTGCCCCGGGAGGTCTCGT AGACCGTGCATCATGAGCACGAATCCTA AACCTCAA		
SAM I riboswitch, *T. tencongensis*	GGTTCTTATCAAGAGAAGCAGAGGGACT GGCCCGACGAAGCTTCAGCAACCGGTG TAATGGCGATCAGCCATGACCAAGGTGC TAAATCCAGCAAGCTCGAACAGCTTGGA AGATAAGAA	SHAPE-Seq v2.0	Figure 5, SI Figure 12
TPP riboswitch, *E. coli*	GGACTCGGGGTGCCCTTCTGCGTGAAG GCTGAGAAATACCCGTATCACCTGATCT GGATAATGCCAGCGTAGGGAAGTTC	SHAPE-Seq v2.0	Figure 5, SI Figure 12

Table S2. RNA folding buffer conditions and ligand concentrations used in replicate experiments.

RNA	Buffer / Ligand	Reference
5S rRNA, *E. coli*	10 mM $MgCl_2$, 100 mM NaCl and 100 mM HEPES (pH 8.0)	(4)
Adenine riboswitch, *V. vulnificus*	10 mM $MgCl_2$, 100 mM NaCl and 100 mM HEPES (pH 8.0), 5μM Ligand	(4)
Cyclic di-GMP riboswitch, *V. cholerae*	10 mM $MgCl_2$, 100 mM NaCl and 100 mM HEPES (pH 8.0), 5μM Ligand	(4)
P4-P6, *Tetrahymena* group I intron ribozyme	10 mM $MgCl_2$, 100 mM NaCl and 100 mM HEPES (pH 8.0)	(4)
RNAse P, specificity domain, *B. subtilis*	10 mM $MgCl_2$, 100 mM NaCl and 100 mM HEPES (pH 8.0)	(2)
$tRNA^{phe}$, *E. coli*	10 mM $MgCl_2$, 100 mM NaCl and 100 mM HEPES (pH 8.0)	(4)
Hepatitis C virus IRES domain	10 mM $MgCl_2$, 100 mM NaCl and 100 mM HEPES (pH 8.0)	(5)
SAM I riboswitch, *T. tencongensis*	10 mM $MgCl_2$, 100 mM NaCl and 100 mM HEPES (pH 8.0), 5μM Ligand	(5)
TPP riboswitch, *E. coli*	10 mM $MgCl_2$, 100 mM NaCl and 100 mM HEPES (pH 8.0), 5μM Ligand	(5)

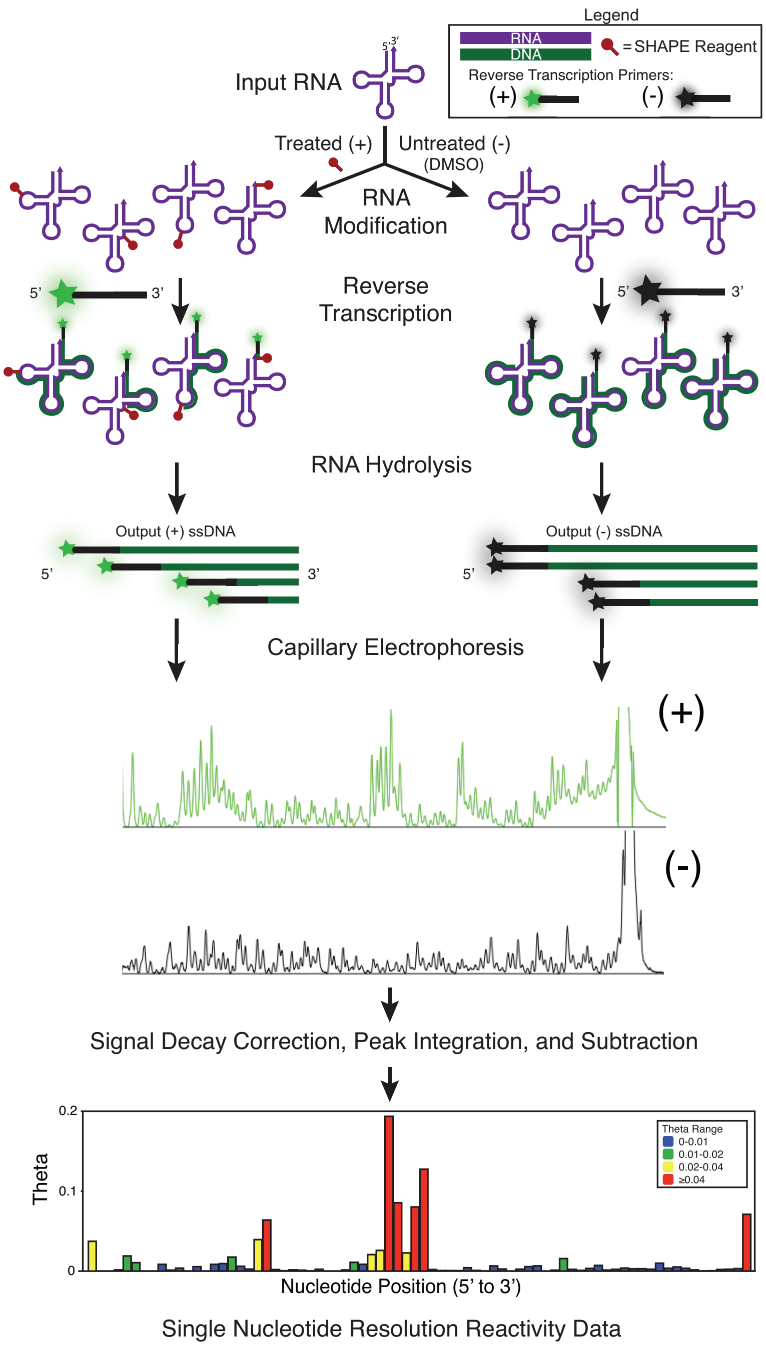

Figure S1. SHAPE-CE Flowchart. Like SHAPE-Seq, SHAPE-CE begins by modifying RNAs with a SHAPE reagent such as 1M7 (1). However, unlike SHAPE-Seq, reverse transcription (RT) is performed with fluorescent primers to create a pool of cDNAs (whose length distribution reflects the distribution of modification positions, like SHAPE-Seq). Two different fluorophores distinguish the modified and control reactions, which are detected with capillary electrophoresis (CE). The resulting CE traces are manually integrated and subtracted to obtain a reactivity spectrum for an RNA. An example with *E. coli* tRNA^{phe} is shown. Not shown are additional di-deoxy-terminated sequencing reactions that are used to align CE peaks to the RNA sequence.

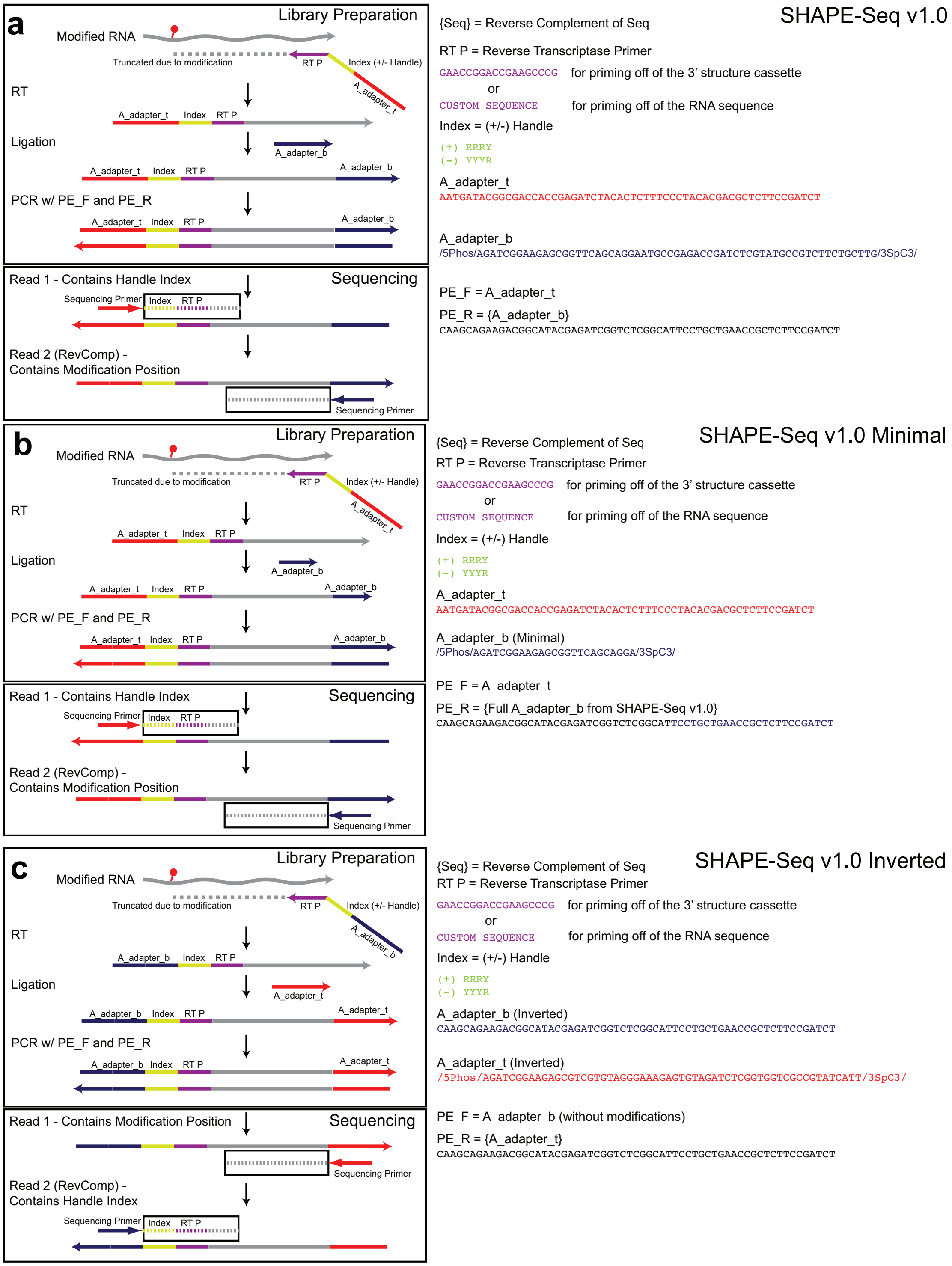

Figure S2. SHAPE-Seq v1.0 and Second Adapter Variation Library Construction Schematics. The left hand side shows adapter and primer orientations for library preparation (top), and which pieces of information are obtained from the library during the sequencing process (bottom), for (a) SHAPE-Seq v1.0, (b) the 'Minimal' adapter configuration, and (c) the 'Inverted' adapter configuration. The right hand side shows DNA sequences of primers and adapters (5' to 3' orientation), color-coded to match the schematic.

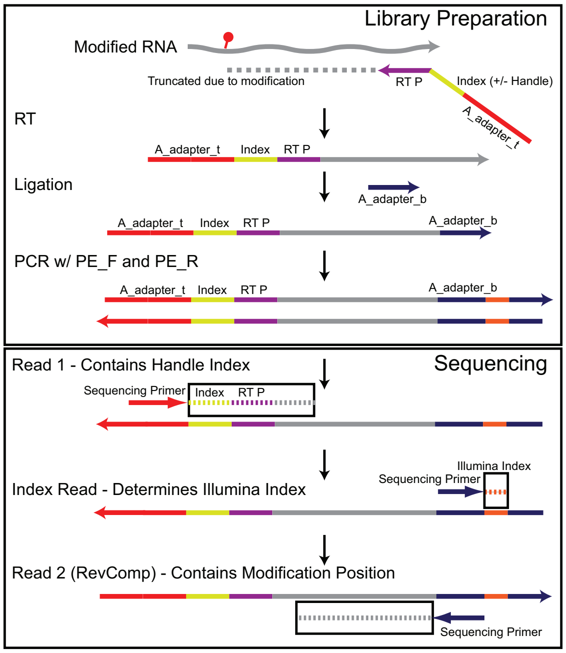

SHAPE-Seq v1.0 Indexed

{Seq} = Reverse Complement of Seq

RT P = Reverse Transcriptase Primer

GAACCGGACCGAAGCCCG for priming off of the 3' structure cassette

or

CUSTOM SEQUENCE for priming off of the RNA sequence

Index = (+/-) Handle

(+) RRRY
(-) YYYR

A_adapter_t
AATGATACGGCGACCACCGAGATCTACACTCTTTCCCTACACGACGCTCTTCCGATCT

A_adapter_b (Indexed)
/5Phos/AGATCGGAAGAGCACACGTCTGAACTCCAGTCAC/3SpC3/

PE_F = A_adapter_t

PE_R = 5' of Illumina Adapter + Illumina Index + {A_adapter_b}
CAAGCAGAAGACGGCATACGAGAT<INDEX SEQUENCE (6nt)>GTGACTGGAGTTCAGACGTGTGCTC

Figure S3. SHAPE-Seq v1.0 library indexing strategy. The left hand side shows adapter and primer orientations for library preparation (top), and which pieces of information are obtained from the library during the sequencing process (bottom) for the SHAPE-Seq v1.0 Indexed library preparation strategy (compare to Figure S2A). The difference between SHAPE-Seq v1.0 and SHAPE-Seq v1.0 Indexed is the presence of Illumina indexing sequences that are added during PCR and sequenced separately during the Index Read. This allows multiple SHAPE-Seq libraries to be sequenced in the same lane following standard Illumina indexing strategies. The right hand side shows DNA sequences of primers and adapters (5' to 3' orientation), color-coded to match the schematic.

SHAPE-Seq v2.0

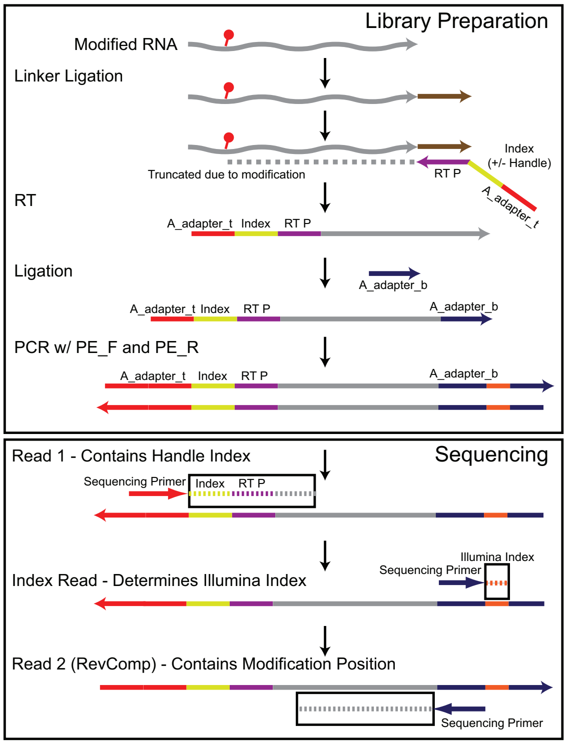

{Seq} = Reverse Complement of Seq

Linker = 5' Adenylated Linker (DNA)
/5rApp/CACTCGGGCACCAAGGA/3ddC/

RT P = Reverse Transcriptase Primer
GTCCTTGGTGCCCGAGTG

Index = (+/-) Handle

(+) RRRY
(-) YYYR

A_adapter_t (v2.0)
CTTTCCCTACACGACGCTCTTCCGATCT

A_adapter_b (Indexed)
/5Phos/AGATCGGAAGAGCACACGTCTGAACTCCAGTCAC/3SpC3/

PE_F = Rest of Illumina Adapter + A_adapter_t
AATGATACGGCGACCACCGAGATCTACACTCTTTCCCTACACGACGCTCTTCCGATCT

PE_R = Rest of Illumina Adapter + Illumina Index + {A_adapter_b}
CAAGCAGAAGACGGCATACGAGAT<INDEX SEQUENCE (6nt)>GTGACTGGAGTTCAGACGTGTGCTC

Figure S4. SHAPE-Seq v2.0 Library Construction Schematic. The left hand side shows adapter and primer orientations for library preparation (top), and which pieces of information are obtained from the library during the sequencing process (bottom) for the Indexed library preparation strategy. The right hand side shows DNA sequences of primers and adapters (5' to 3' orientation), color-coded to match the schematic. SHAPE-Seq v2.0 uses a 'universal' RT priming strategy as well as the standard Illumina library indexing strategy.

Table S3. List of barcoded reverse transcription primers used during the SHAPE-Seq v2.0 library generation. Illumina adapter sequences are in black, the (+/-) handles are in green, internal barcodes are in red, and the RT priming sites are in purple. Illumina TruSeq indexes can be found at:
http://supportres.illumina.com/documents/myillumina/6378de81-c0cc-47d0-9281-724878bb1c30/2012-09-18_illuminacustomersequenceletter.pdf

RNA	RT Primer Sequences	Illumina Index# (TruSeq)
tRNA^{phe}	/5Biosg/CTTTCCCTACACGACGCTCTTCCGATCTYYYRgtccttggtgcccgagtg<br>/5Biosg/CTTTCCCTACACGACGCTCTTCCGATCTRRRYgtccttggtgcccgagtg	1,2,3
Adenine riboswitch, *V. vulnificus*	/5Biosg/CTTTCCCTACACGACGCTCTTCCGATCTYYYRgtccttggtgcccgagtg<br>/5Biosg/CTTTCCCTACACGACGCTCTTCCGATCTRRRYgtccttggtgcccgagtg	4,5,6
5S rRNA, *E. coli*	/5Biosg/CTTTCCCTACACGACGCTCTTCCGATCTYYYRgtccttggtgcccgagtg<br>/5Biosg/CTTTCCCTACACGACGCTCTTCCGATCTRRRYgtccttggtgcccgagtg	1,2,3
P4-P6, *Tetrahymena* group I intron ribozyme	/5Biosg/CTTTCCCTACACGACGCTCTTCCGATCTYYYRCCgtccttggtgcccgagtg<br>/5Biosg/CTTTCCCTACACGACGCTCTTCCGATCTRRRYCCgtccttggtgcccgagtg	1,2,3
RNAse P, specificity domain, *B. subtilis*	/5Biosg/CTTTCCCTACACGACGCTCTTCCGATCTYYYRAgtccttggtgcccgagtg<br>/5Biosg/CTTTCCCTACACGACGCTCTTCCGATCTRRRYAgtccttggtgcccgagtg	4,5,6
TPP riboswitch, *E. coli*	/5Biosg/CTTTCCCTACACGACGCTCTTCCGATCTYYYRAgtccttggtgcccgagtg<br>/5Biosg/CTTTCCCTACACGACGCTCTTCCGATCTRRRYAgtccttggtgcccgagtg	4,5,6
SAM I riboswitch, *T. tencongensis*	/5Biosg/CTTTCCCTACACGACGCTCTTCCGATCTYYYRAgtccttggtgcccgagtg<br>/5Biosg/CTTTCCCTACACGACGCTCTTCCGATCTRRRYAgtccttggtgcccgagtg	10,11,12
Cyclic di-GMP riboswitch, *V. cholerae*	/5Biosg/CTTTCCCTACACGACGCTCTTCCGATCTYYYRCCgtccttggtgcccgagtg<br>/5Biosg/CTTTCCCTACACGACGCTCTTCCGATCTRRRYCCgtccttggtgcccgagtg	7,8,9
Hepatitis C virus IRES domain	/5Biosg/CTTTCCCTACACGACGCTCTTCCGATCTYYYRCCgtccttggtgcccgagtg<br>/5Biosg/CTTTCCCTACACGACGCTCTTCCGATCTRRRYCCgtccttggtgcccgagtg	10,11,12

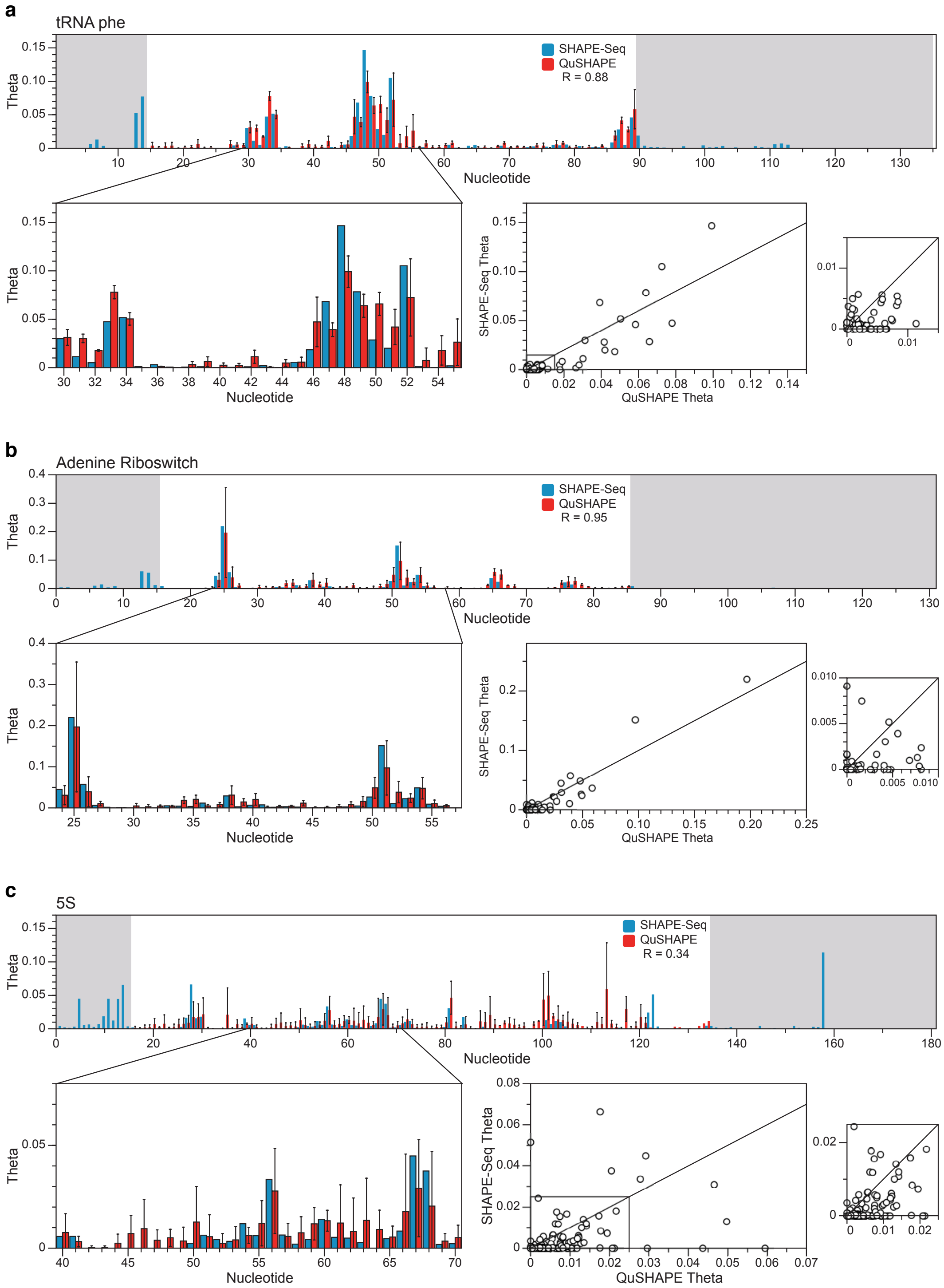
a
tRNA phe
SHAPE-Seq
QuSHAPE
R = 0.88
Theta
Nucleotide
SHAPE-Seq Theta
QuSHAPE Theta
b
Adenine Riboswitch
SHAPE-Seq
QuSHAPE
R = 0.95
Theta
Nucleotide
SHAPE-Seq Theta
QuSHAPE Theta
c
5S
SHAPE-Seq
QuSHAPE
R = 0.34
Theta
Nucleotide
SHAPE-Seq Theta
QuSHAPE Theta

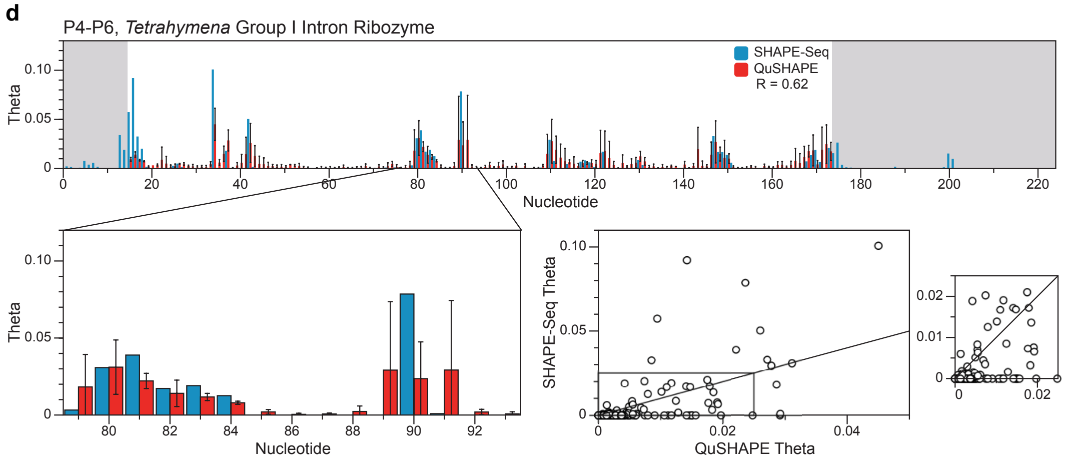

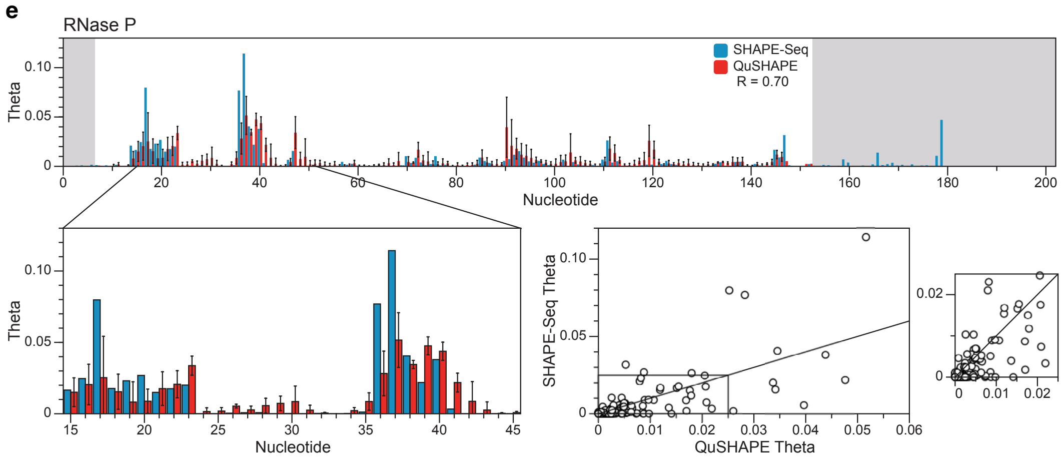

Figure S5. QuSHAPE vs. SHAPE-Seq (v1.0) detailed comparisons. For each RNA, SHAPE-Seq and QuSHAPE θ's are plotted on top, with a zoomed window on portions of the comparison shown on the bottom left. The bottom right shows SHAPE-Seq vs. QuSHAPE θ's plotted as a scatter plot, from which the Pearson's correlation (R) between the two techniques is calculated. Gray boxes represent regions for which no QuSHAPE data is available. Due to difficulties encountered in the alignment step of the QuSHAPE data analysis pipeline, we often found that a single QuSHAPE experiment yielded only a fraction of the reactivities for an individual RNA. To remedy this, we performed replicate QuSHAPE experiments for each RNA, and calculated the average and standard deviation of the QuSHAPE reactivities for each nucleotide position. These QuSHAPE reactivities were then converted to QuSHAPE θ's by dividing by a normalization factor so that they summed to 1 over the range of nucleotides for which reactivity data was obtained. Overall, there was a strong degree of correlation between the two methods for each of the RNAs. Specifically, the Pearson correlations between θ's for each RNA were: 0.88 ($tRNA^{phe}$) 0.70 (RNase P), 0.62 (ribozyme) and 0.95 (adenine riboswitch aptamer). Only the historically difficult 5S rRNA had a poor correlation (0.34).

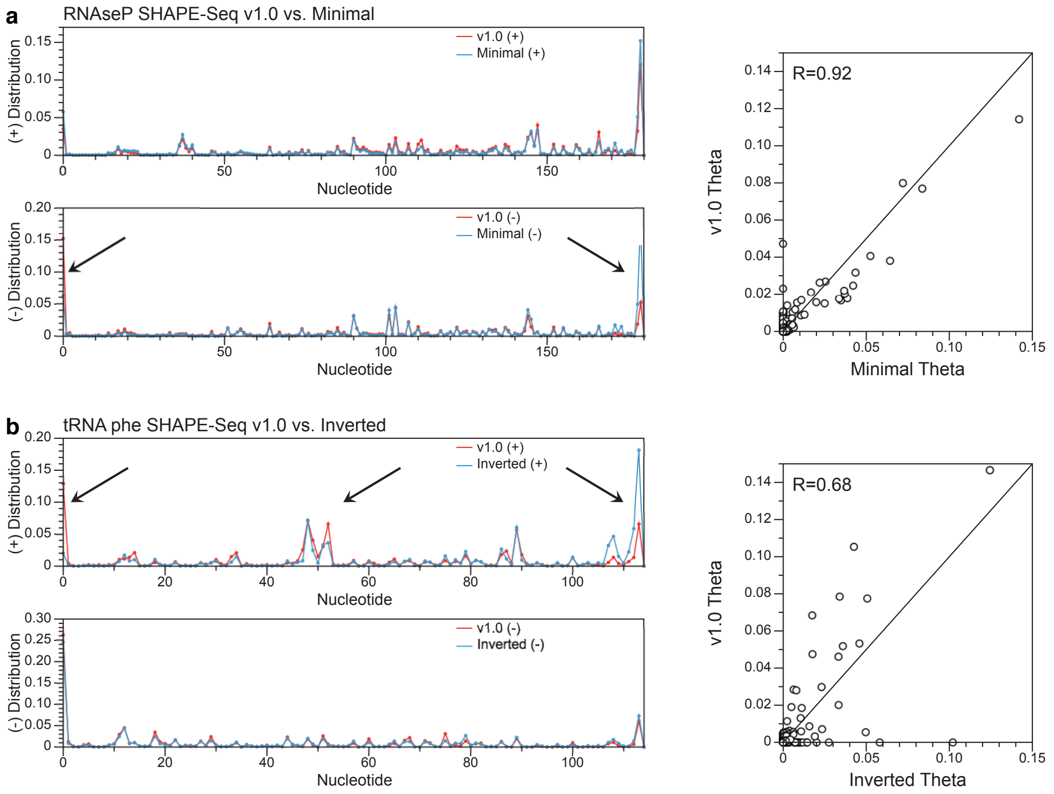

Figure S6. SHAPE-Seq v1.0 vs. Minimal or Inverted adapter variations for RNase P and tRNA. (+) and (-) fragment distributions are plotted for (a) v1.0 vs. Minimal for RNase P, and for (b) v1.0 vs. Inverted for tRNA. Pearson correlation values for these comparisons are summarized in Figure 2. Arrows denote specific places of discrepancy discussed in the text. Scatter plots show θ value comparisons between the two libraries for the RNAs with Pearson correlation values shown in the plots.

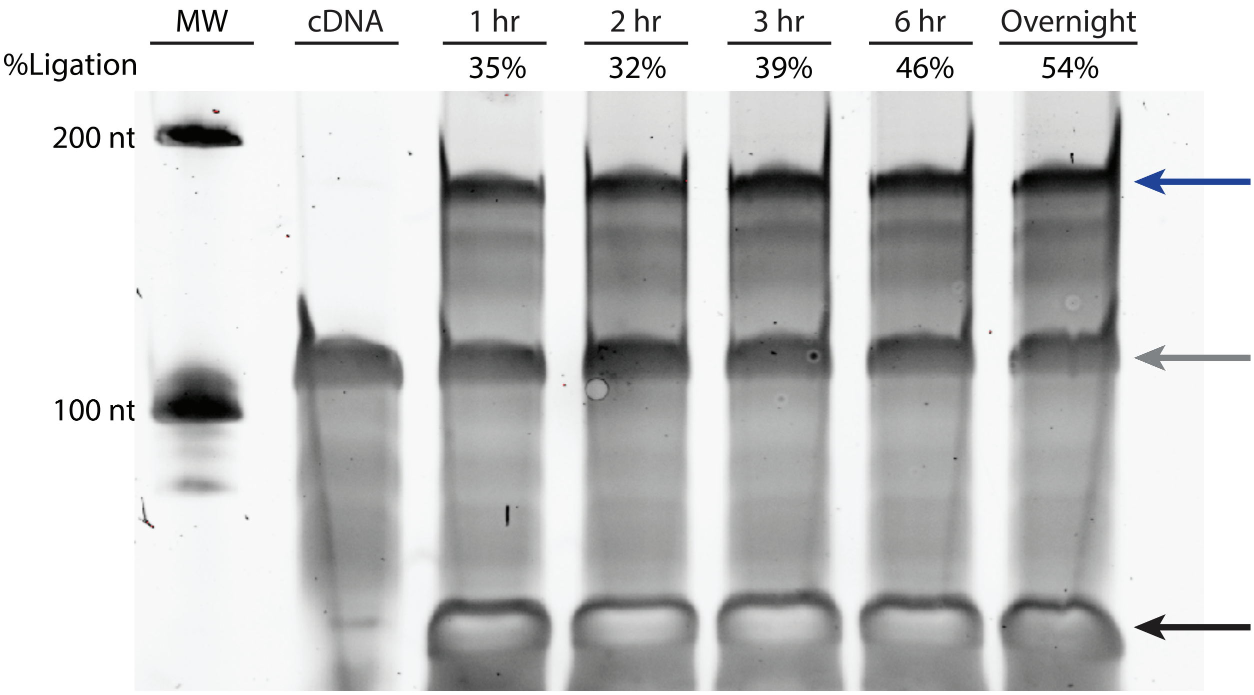

Figure S7. Time course of CircLigase I ligation efficiency. A 126 nt cDNA (gray arrow) was ligated to the 61nt SHAPE-Seq v1.0 Illumina adapter (Figure S1, black arrow) for one, two, three, or six hours or overnight at 68 °C to generate a 187 nt ligation product (blue arrow). Slight ligation improvement is observable over time by integration of the disappearance of the primer band. Note, however, that the gel is stained with SYBR and only provides general trends and is not absolutely quantitative. Ligation was halted after 2 hrs because the improvement gained by further incubation was less important relative to the increase in protocol time.

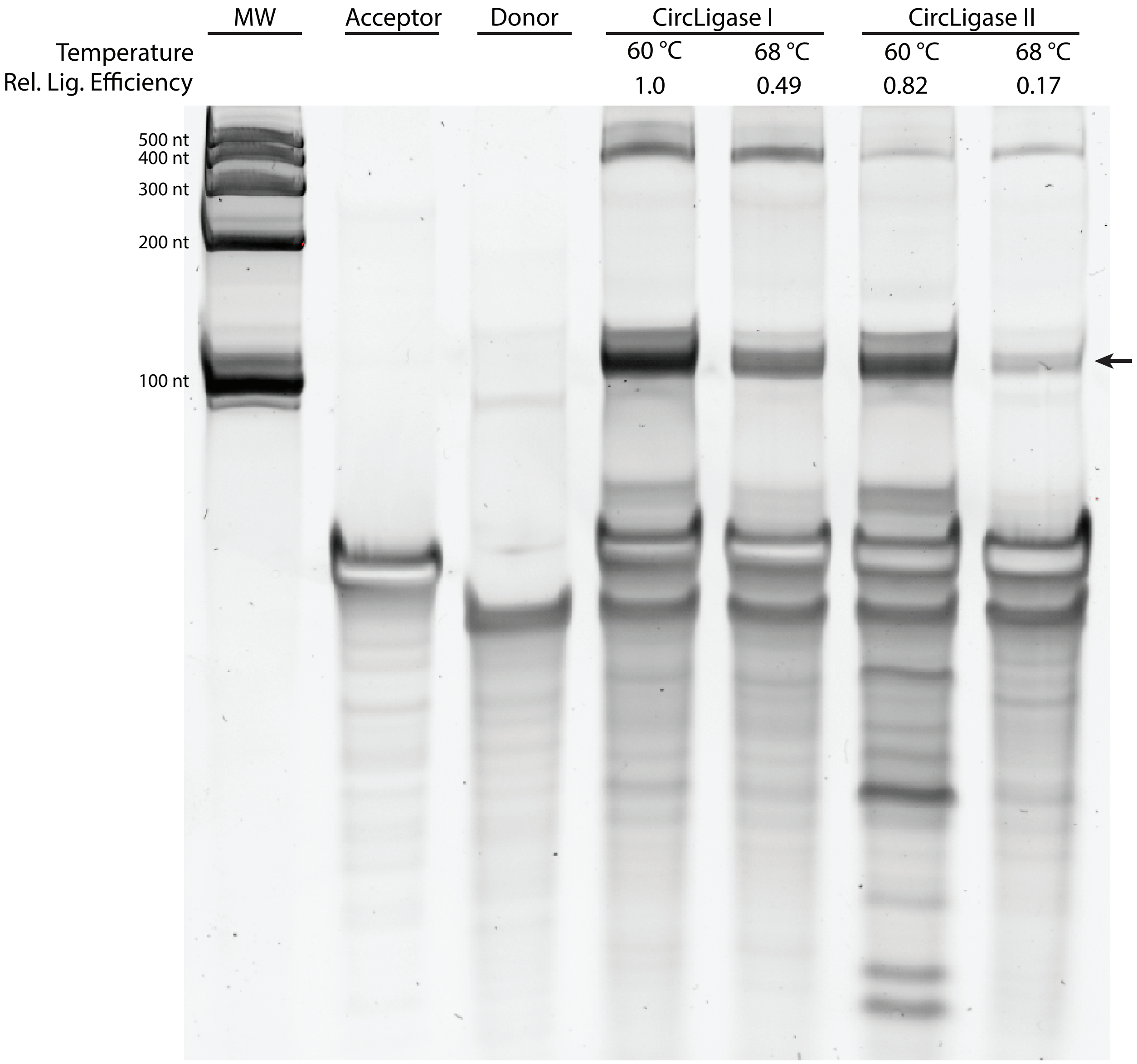

Figure S8. Ligase comparison for addition of SHAPE-Seq second adapter. CircLigase I and CircLigase II (Epicentre) were used to ligate the 50 nt RT primer (donor) to the 61 nt Illumina adapter (acceptor) at both 68 °C and 60 °C (manufacturer's suggestion) for two hours. The expected ligation product is denoted by the black arrow (101 nt). We found the optimum ligation condition to be CircLigase I at 60 °C for two hours by determining the relative intensity of the ligated product band. Note, however, that the gel is stained with SYBR gold and is not absolutely quantitative.

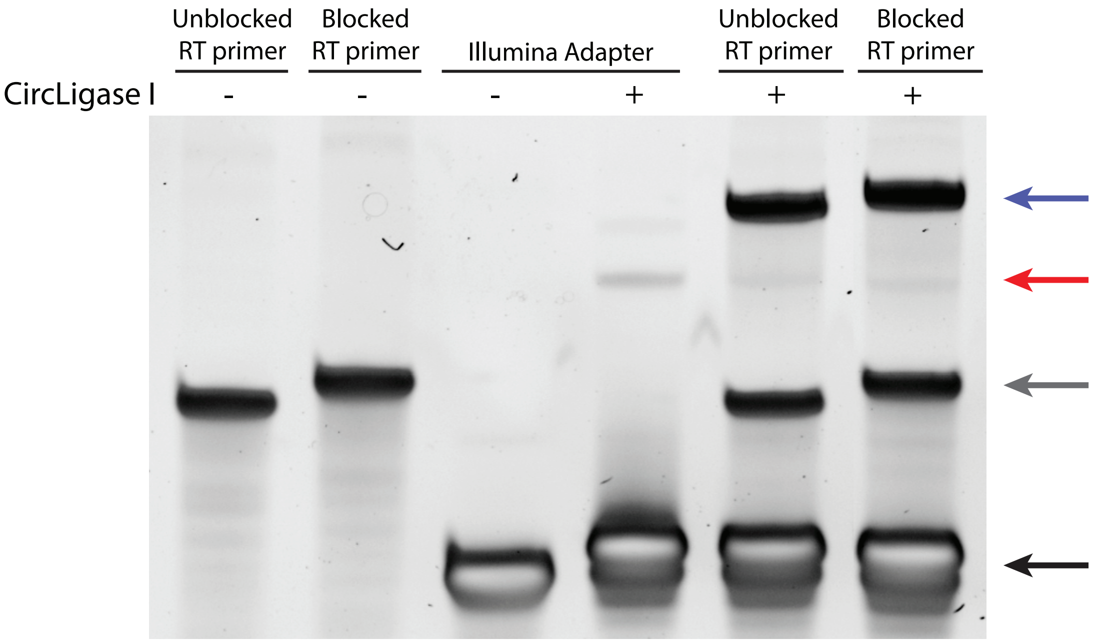

Figure S9. Modification and optimization of RT primer blocking groups for adapter ligation. Comparison of effect of blocking groups on the possible formation of RT primer concatemers. The top (blue) arrow shows the successfully ligated RT primer (gray arrow) + adapter (black arrow) (60 °C for 2 hours with Circligase I). The red arrow marks the location of adapter dimer formation (studied further below in Figure S9). No bands corresponding to RT primer concatemers were observed.

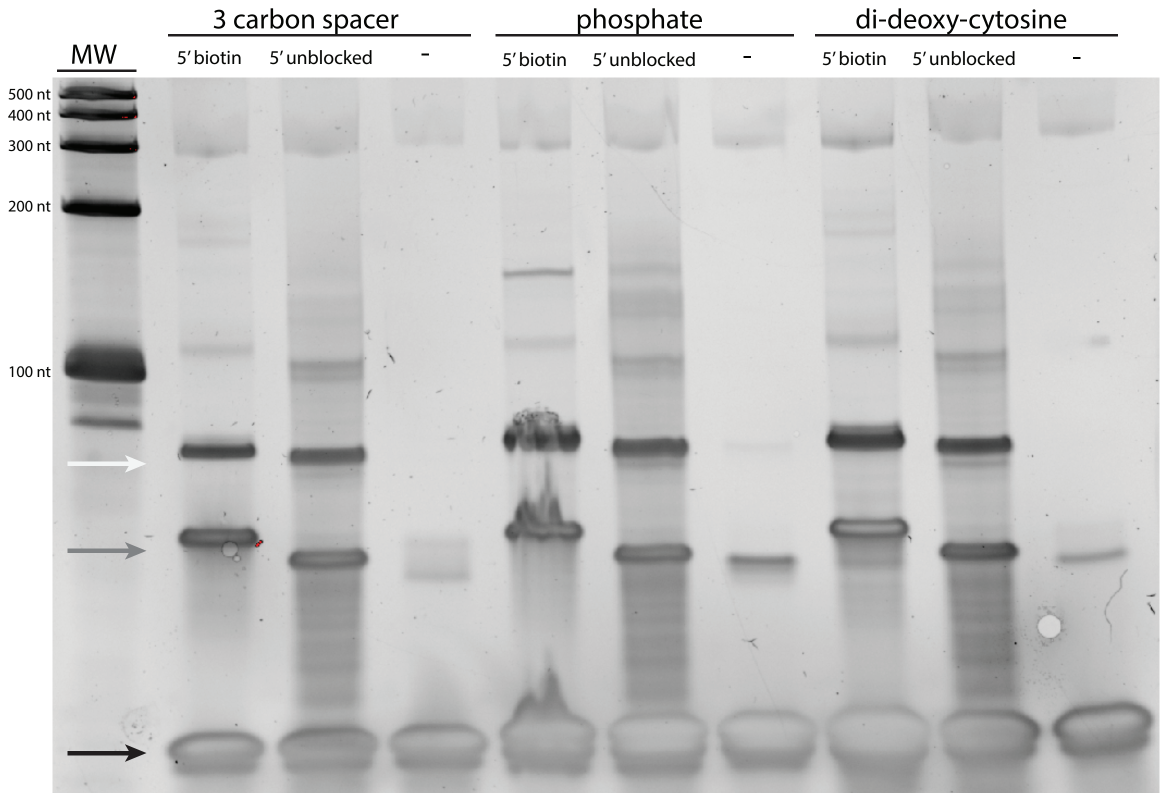

Figure S10. Effect of 3' blocking group on second adapter concatemerization and ligation efficiency. Three modifications (3 carbon spacer, phosphate, and di-deoxy-cytosine) were added to the 3' end of the adapter (black arrow), which was then ligated (60 °C for 2 hours with Circligase I) to an RT primer (gray arrow) that was either 5' blocked (57 nt) with biotin or unblocked (54 nt). A third control lane for each 3' modification consisting of the ligation of the adapter without RT primer is shown (-). Regardless of 3' modification, some adapter form concatemers during the ligation, with concatemers showing up at 50 nt, near the size of the RT primers (gray arrow). However, this effect is the weakest for the 3 carbon spacer modification, which also shows the least amount of adapter concatemer ligated to the RT primer, seen as bands longer than the expected ligated product which is 79 nt or 82 nt (white arrow). While no modification stood out as the clear best, the 3' 3-carbon spacer modification was chosen for its relative cleanliness and lower cost than di-deoxy-cytosine.

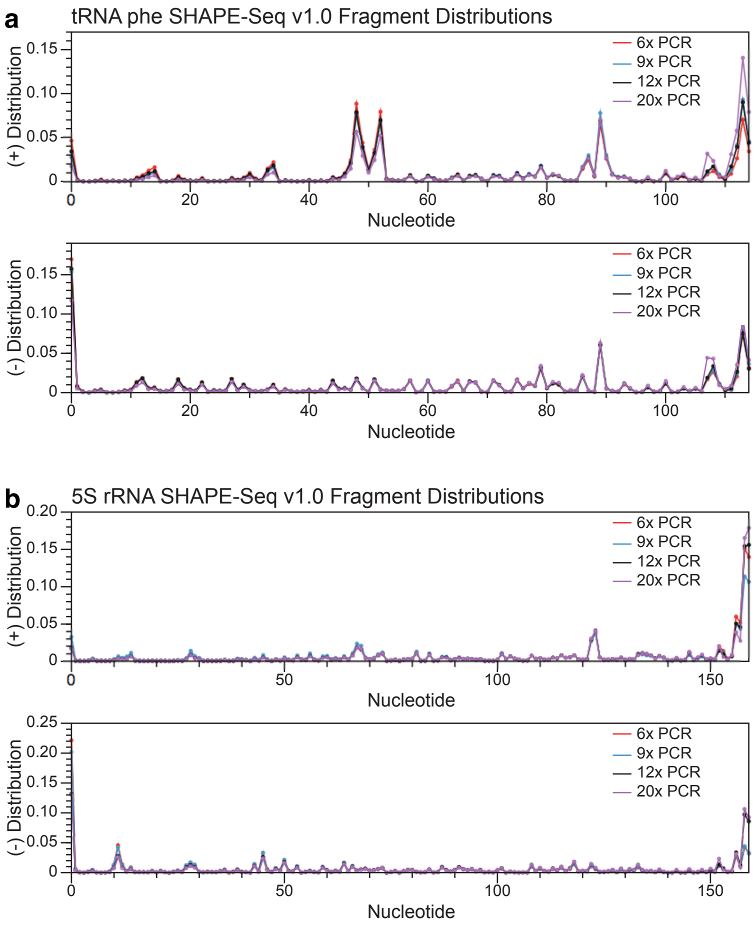

Figure S11. SHAPE-Seq v1.0 fragment distributions for different numbers of PCR cycles. (+) and (-) fragment distributions are plotted for SHAPE-Seq v1.0 with varying numbers of PCR cycles for (a) tRNA^{phe} and (b) 5S rRNA (see Figure 4). Pearson correlation values for specific comparisons are shown in Figure 3.

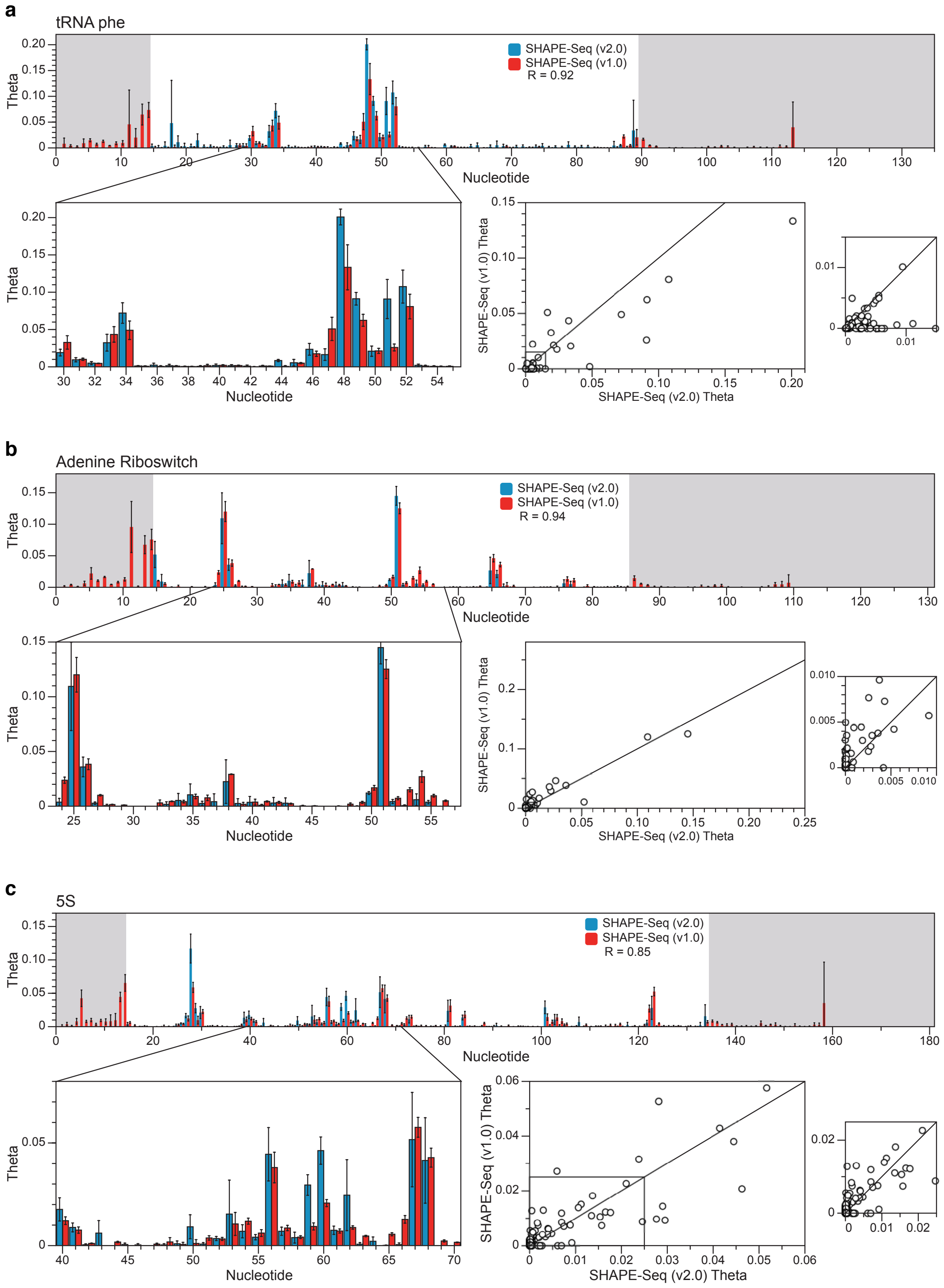

a
tRNA phe
SHAPE-Seq (v2.0)
SHAPE-Seq (v1.0)
R = 0.92
Theta
Nucleotide
SHAPE-Seq (v1.0) Theta
SHAPE-Seq (v2.0) Theta
b
Adenine Riboswitch
SHAPE-Seq (v2.0)
SHAPE-Seq (v1.0)
R = 0.94
Theta
Nucleotide
SHAPE-Seq (v1.0) Theta
SHAPE-Seq (v2.0) Theta
c
5S
SHAPE-Seq (v2.0)
SHAPE-Seq (v1.0)
R = 0.85
Theta
Nucleotide
SHAPE-Seq (v1.0) Theta
SHAPE-Seq (v2.0) Theta

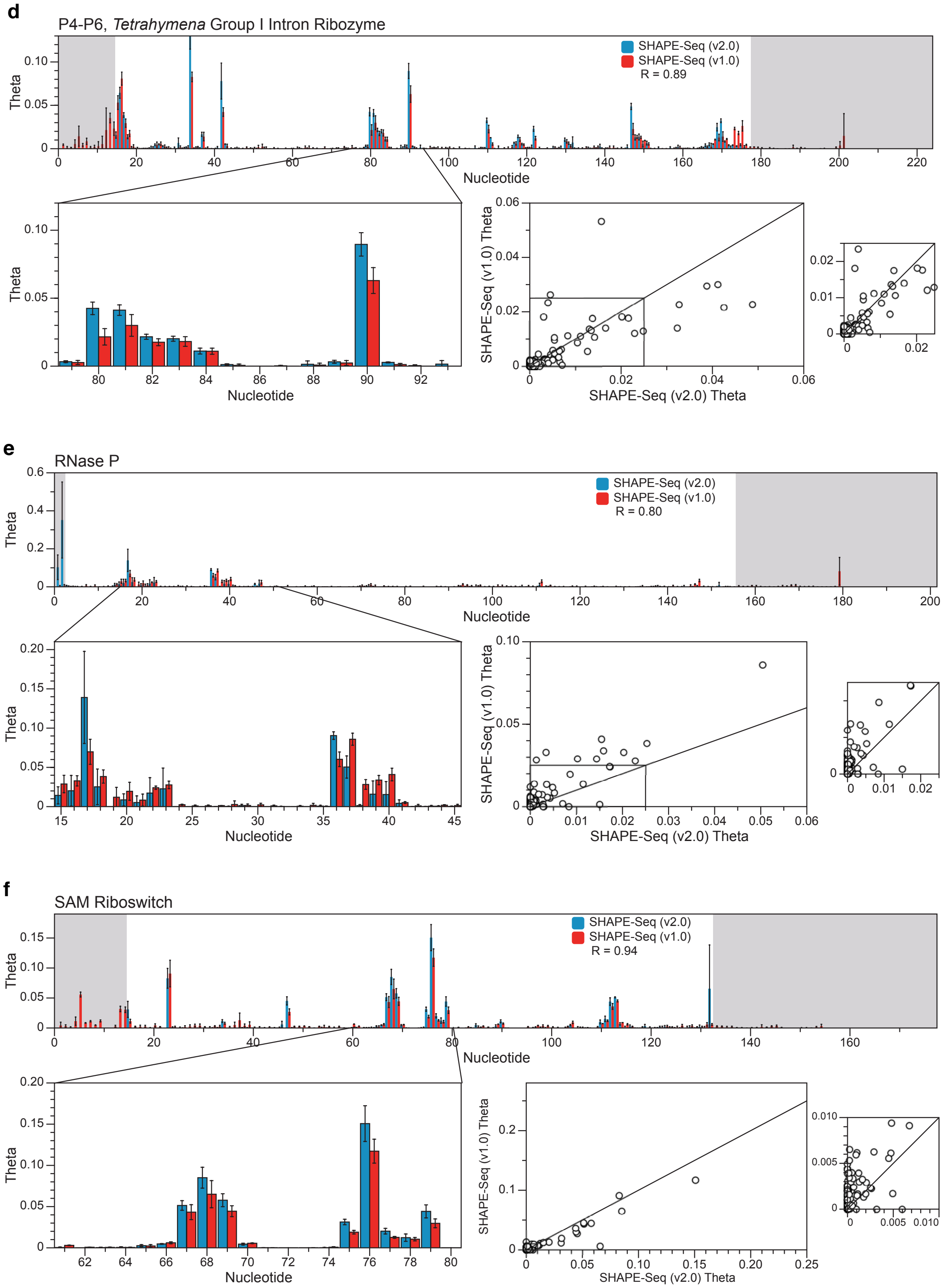
d
P4-P6, Tetrahymena Group I Intron Ribozyme
SHAPE-Seq (v2.0)
SHAPE-Seq (v1.0)
R = 0.89
Theta
Nucleotide
SHAPE-Seq (v1.0) Theta
SHAPE-Seq (v2.0) Theta
e
RNase P
SHAPE-Seq (v2.0)
SHAPE-Seq (v1.0)
R = 0.80
Theta
Nucleotide
SHAPE-Seq (v1.0) Theta
SHAPE-Seq (v2.0) Theta
f
SAM Riboswitch
SHAPE-Seq (v2.0)
SHAPE-Seq (v1.0)
R = 0.94
Theta
Nucleotide
SHAPE-Seq (v1.0) Theta
SHAPE-Seq (v2.0) Theta

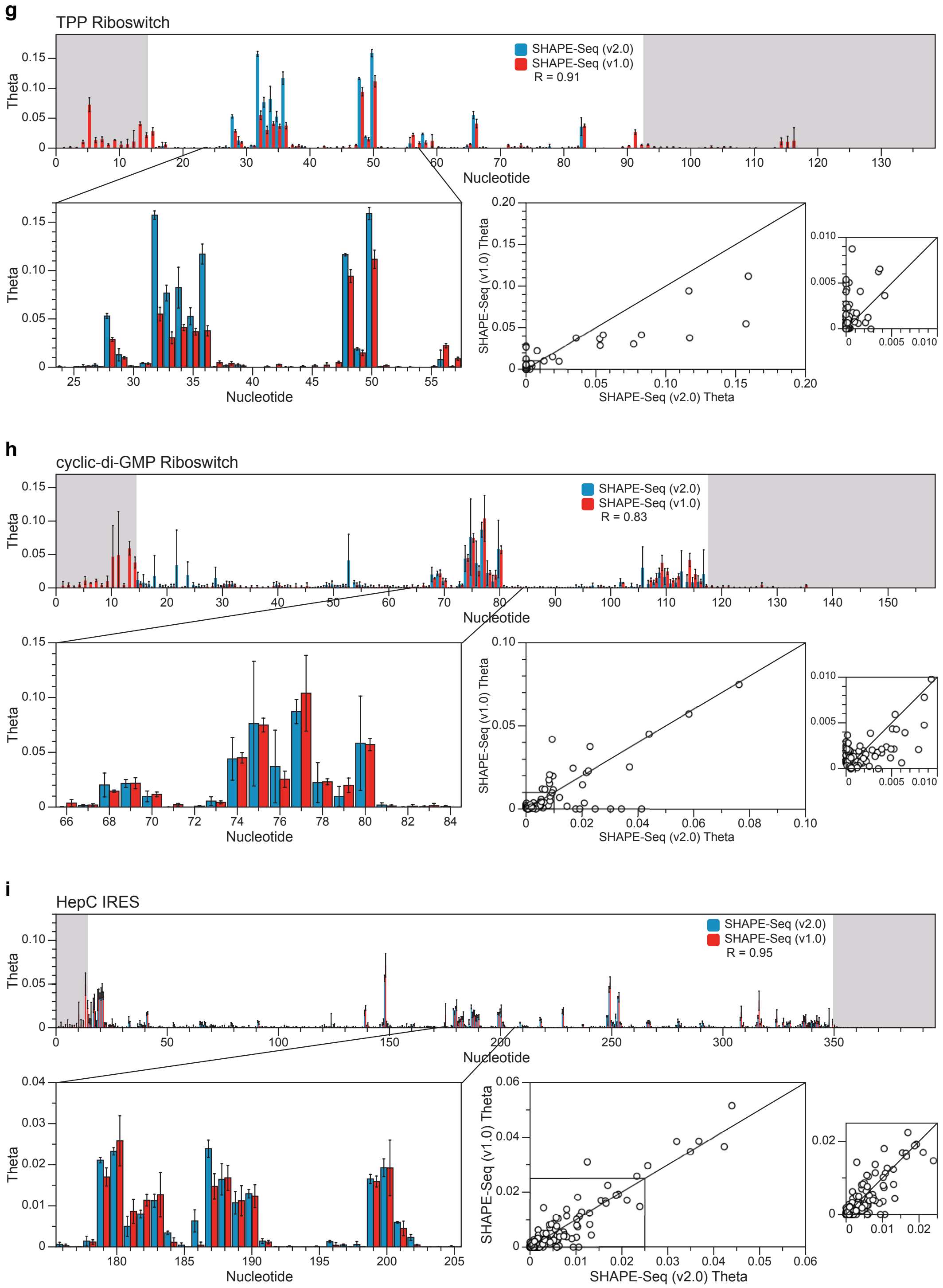

Figure S12. SHAPE-Seq v2.0 vs. SHAPE-Seq v1.0. For each RNA, SHAPE-Seq v2.0 (blue) and SHAPE-Seq v1.0 (red) θ's are plotted on top, with a zoomed window on portions of the comparison shown on the bottom left. Error bars are calculated as standard deviations of reactivities at each nucleotide from three independent replicate experiments. The bottom right shows SHAPE-Seq v2.0 vs. v1.0 θ's plotted as a scatter plot, from which the Pearson's

correlation (R) between the two techniques is calculated. Gray boxes represent flanking structure cassette regions included in the RNAs for SHAPE-Seq v1.0, but not present in the RNAs for SHAPE-Seq v2.0 (see Table S1).

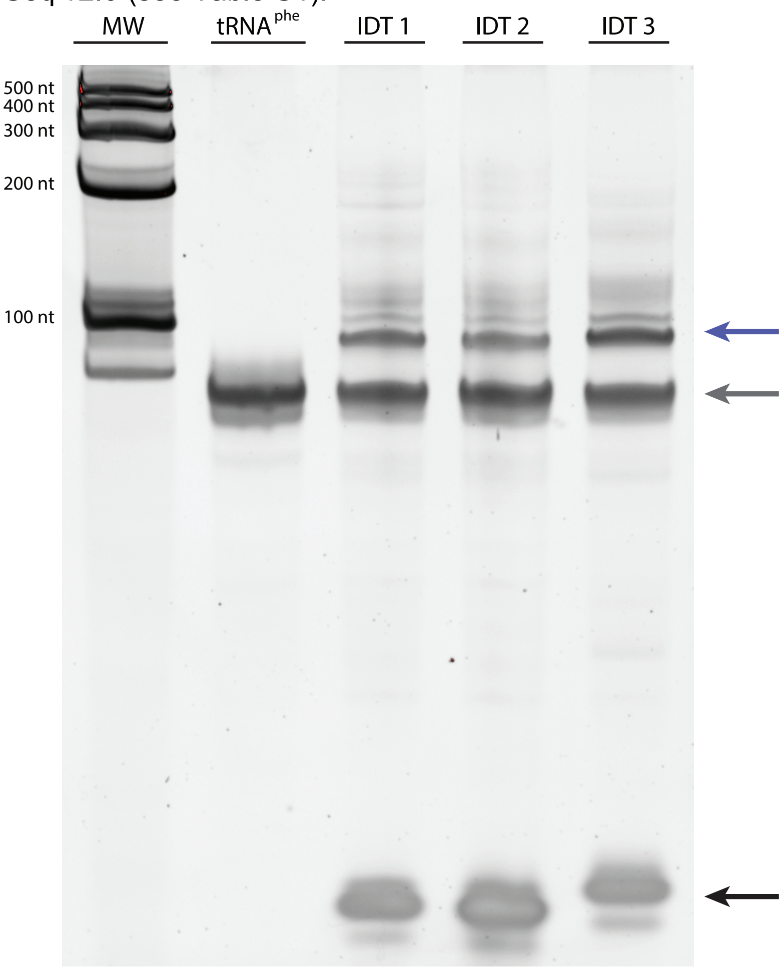

Figure S13. Choice of 5' adenylated linker sequence for SHAPE-Seq v2.0 universal priming strategy. Each linker choice was ligated onto an unmodified strand of $tRNA^{phe}$ using T4 RNA Ligase 2, truncated KQ overnight at room temperature. The gray arrow indicates the bands that correspond to the full-length, unligated tRNA, the blue arrow above shows the bands corresponding to the successfully ligated tRNA+linker, and the bands at the very bottom of the figure correspond to unligated linker. IDT1, IDT2 and IDT3 sequences are commercially available from Integrated DNA Technologies, Inc. at: http://www.idtdna.com/pages/products/mirna/mirna-cloning-products. IDT2 was chosen due to it having the highest melting temperature with its RT primer (Figure S4).

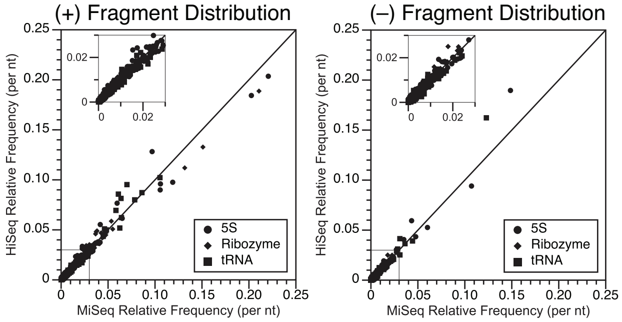

R values	5S rRNA	*Tetrahymena* Ribozyme	$tRNA^{phe}$	All
(+) Fragments	0.99	0.99	1.0	0.99
(–) Fragments	0.99	1.0	1.0	1.0

Figure S14. SHAPE-Seq v2.0 reactivities generated from the MiSeq and HiSeq platforms. (+) and (-) fragment distributions for each RNA were compared between sequencing platforms as in Figure 2. Pearson correlation values for individual comparisons between fragment distributions, are shown on the bottom.

Table S4. RNA structure prediction accuracy using the RNAStructure (6) *fold* algorithm with no SHAPE-Seq constraints.

RNA	Sensitivity	PPV
5S rRNA	10/35=28.6%	10/40=25.0%
Adenine Riboswitch	21/21=100%	21/21=100%
Cyclic di-GMP riboswitch	21/28=75.0%	21/27=77.8%
P4-P6, *Tetrahymena* group I intron ribozyme	46/48=95.8%	47/55=85.5%
RNAse P	23/42=54.8%	23/46=50.0%
$tRNA^{phe}$	20/21= 95.2%	20/20=100%
Hepatitis C IRES	41/104=39.4%	41/108=38.0%
SAM I riboswitch	29/39=74.4%	29/36=80.6%
TPP riboswitch	17/22=77.3%	17/20=85.0%
Total	228/360=63.3%	229/373=61.4%

Table S5. RNA structure prediction accuracy using SHAPE-Seq v2.0 reactivity data (ρ's) as constraints in the RNAStructure (6) *fold* algorithm with *m* = 1.8 and *b* = -0.6.

RNA	Sensitivity	PPV
5S rRNA	34/35=97.1%	34/37=91.9%
Adenine Riboswitch	21/21=100%	21/21=100%
Cyclic di-GMP riboswitch	19/28=67.9%	19/24=79.2%
P4-P6, *Tetrahymena* group I intron ribozyme	45/48=93.8%	46/51=90.2%
RNAse P	22/42=52.4%	22/44=50.0%
$tRNA^{phe}$	21/21= 100%	21/21=100%
Hepatitis C IRES	81/104=77.9%	81/96=84.4%
SAM I riboswitch	32/39=82.1%	32/37=86.5%
TPP riboswitch	17/22=77.3%	17/20=85.0%
Total	292/360=81.1%	293/351=83.5%

Table S6. RNA structure prediction accuracy using SHAPE-Seq v2.0 reactivity data (ρ's) as constraints in the RNAStructure (6) *fold* algorithm with *m* = 1.1 and *b* = -0.3.

RNA	Sensitivity	PPV
5S rRNA	34/35=97.1%	34/37=91.9%
Adenine Riboswitch	21/21=100%	21/21=100%
Cyclic di-GMP riboswitch	21/28=75.0%	21/28=75.0%
P4-P6, *Tetrahymena* group I intron ribozyme	44/48=91.7%	45/49=91.8%
RNAse P	33/42=78.6%	33/40=82.5%
$tRNA^{phe}$	20/21= 95.2%	20/20=100%
Hepatitis C IRES	81/104=77.9%	81/90=90.0%
SAM I riboswitch	32/39=82.1%	32/37=86.5%
TPP riboswitch	17/22=77.3%	17/20=85.0%
Total	303/360=84.2%	304/342=88.9%

Table S7. Data deposition table. SHAPE-Seq reactivity spectra generated in this work are freely available from the RNA Mapping Database (RMDB) (7) (http://rmdb.stanford.edu/repository/), accessible using the RMDB ID numbers indicated below.

Name	Library Type	RMDB ID	Figure
5S rRNA, *E. coli*	SHAPE-Seq v1.0	5SSC_1M7_0001	Figure 2, SI Figure 5,
5S rRNA, *E. coli*	SHAPE-Seq v1.0 Minimal	5SSC_1M7_0002	Figure 2
5S rRNA, *E. coli*	SHAPE-Seq v1.0 Inverted	5SSC_1M7_0003	Figure 2
5S rRNA, *E. coli*	SHAPE-Seq v1.0 Indexed Replicates 1-3	5SSC_1M7_0004 5SSC_1M7_0005, 5SSC_1M7_0006	Figure 3, Figure 5, SI Figure 11, SI Figure 12
5S rRNA, *E. coli*	SHAPE-Seq v2.0 Indexed Replicates 1-3	5SRRNA_1M7_0001, 5SRRNA_1M7_0002, 5SRRNA_1M7_0003	Figure 5, SI Figure 12, SI Figure 14
Adenine riboswitch, *V. vulnificus*	SHAPE-Seq v1.0	ADDSC_1M7_0001	Figure 2, SI Figure 5,
Adenine riboswitch, *V. vulnificus*	SHAPE-Seq v1.0 Minimal	ADDSC_1M7_0002	Figure 2
Adenine riboswitch, *V. vulnificus*	SHAPE-Seq v1.0 Inverted	ADDSC_1M7_0003	Figure 2
Adenine riboswitch, *V. vulnificus*	SHAPE-Seq v1.0 Indexed Replicates 1-3	ADDSC_1M7_0004 ADDSC_1M7_0005, ADDSC_1M7_0006	Figure 5, SI Figure 12
Adenine riboswitch, *V. vulnificus*	SHAPE-Seq v2.0 Indexed Replicates 1-3	ADDRSW_1M7_0001, ADDRSW_1M7_0002, ADDRSW_1M7_0003	Figure 5, SI Figure 12
Cyclic di-GMP riboswitch, *V. cholerae*	SHAPE-Seq v1.0 Indexed Replicates 1-3	GMPSC_1M7_0001 GMPSC_1M7_0002, GMPSC_1M7_0003	Figure 5, SI Figure 12
Cyclic di-GMP riboswitch, *V. cholerae*	SHAPE-Seq v2.0 Indexed Replicates 1-3	CIDGMP_1M7_0001, CIDGMP_1M7_0002, CIDGMP_1M7_0003	Figure 5, SI Figure 12
P4-P6, *Tetrahymena* group I intron ribozyme	SHAPE-Seq v1.0	TRIBSC_1M7_0001	Figure 2, SI Figure 5,
P4-P6, *Tetrahymena* group I intron ribozyme	SHAPE-Seq v1.0 Minimal	TRIBSC_1M7_0002	Figure 2
P4-P6, *Tetrahymena* group I intron ribozyme	SHAPE-Seq v1.0 Inverted	TRIBSC_1M7_0003	Figure 2
P4-P6, *Tetrahymena* group I intron ribozyme	SHAPE-Seq v1.0 Indexed Replicates 1-3	TRIBSC_1M7_0004 TRIBSC_1M7_0005, TRIBSC_1M7_0006	Figure 5, SI Figure 12
P4-P6, *Tetrahymena* group I intron ribozyme	SHAPE-Seq v2.0 Indexed Replicates 1-3	TRP4P6_1M7_0001, TRP4P6_1M7_0002, TRP4P6_1M7_0003	Figure 5, SI Figure 12, SI Figure 14
RNAse P, specificity domain, *B. subtilis*	SHAPE-Seq v1.0	RNPSC_1M7_0001	Figure 2, SI Figure 5, SI Figure 6
RNAse P, specificity domain, *B. subtilis*	SHAPE-Seq v1.0 Minimal	RNPSC_1M7_0002	Figure 2, SI Figure 6
RNAse P, specificity domain, *B. subtilis*	SHAPE-Seq v1.0 Inverted	RNPSC_1M7_0003	Figure 2, SI Figure 6
RNAse P, specificity domain, *B. subtilis*	SHAPE-Seq v1.0 Indexed Replicates 1-3	RNPSC_1M7_0004 RNPSC_1M7_0005, RNPSC_1M7_0006	Figure 5, SI Figure 12

RNAse P, specificity domain, *B. subtilis*	SHAPE-Seq v2.0 Indexed Replicates 1-3	RNASEP_1M7_0001, RNASEP_1M7_0002, RNASEP_1M7_0003	Figure 5, SI Figure 12
tRNA^{phe}, *E. coli*	SHAPE-Seq v1.0	TRNASC_1M7_0001	Figure 2, SI Figure 5 SI Figure 6
tRNA^{phe}, *E. coli*	SHAPE-Seq v1.0 Minimal	TRNASC_1M7_0002	Figure 2, SI Figure 6
tRNA^{phe}, *E. coli*	SHAPE-Seq v1.0 Inverted	TRNASC_1M7_0003	Figure 2, SI Figure 6
tRNA^{phe}, *E. coli*	SHAPE-Seq v1.0 Indexed Replicates 1-3	TRNASC_1M7_0004 TRNASC_1M7_0005, TRNASC_1M7_0006	Figure 5, SI Figure 12
tRNA^{phe}, *E. coli*	SHAPE-Seq v2.0 Indexed Replicates 1-3	TRNAPH_1M7_0001, TRNAPH_1M7_0002, TRNAPH_1M7_0003	Figure 3, Figure 5, SI Figure 11 SI Figure 12, SI Figure 14
Hepatitis C virus IRES domain	SHAPE-Seq v1.0 Indexed Replicates 1-3	HEPCSC_1M7_0001 HEPCSC_1M7_0002, HEPCSC_1M7_0003	Figure 5, SI Figure 12
Hepatitis C virus IRES domain	SHAPE-Seq v2.0 Indexed Replicates 1-3	HCIRES_1M7_0001, HCIRES_1M7_0002, HCIRES_1M7_0003	Figure 5, SI Figure 12
SAM I riboswitch, *T. tencongensis*	SHAPE-Seq v1.0 Indexed Replicates 1-3	SAMSC_1M7_0001 SAMSC_1M7_0002, SAMSC_1M7_0003	Figure 5, SI Figure 12
SAM I riboswitch, *T. tencongensis*	SHAPE-Seq v2.0 Indexed Replicates 1-3	SAMRSW_1M7_0001, SAMRSW_1M7_0002, SAMRSW_1M7_0003	Figure 5, SI Figure 12
TPP riboswitch, *E. coli*	SHAPE-Seq v1.0 Indexed Replicates 1-3	TPPSC_1M7_0001, TPPSC_1M7_0002, TPPSC_1M7_0003	Figure 5, SI Figure 12
TPP riboswitch, *E. coli*	SHAPE-Seq v2.0 Indexed Replicates 1-3	TPPRSW_1M7_0001, TPPRSW_1M7_0002, TPPRSW_1M7_0003	Figure 5, SI Figure 12

References

1. Merino, E.J., Wilkinson, K.A., Coughlan, J.L. and Weeks, K.M. (2005) RNA structure analysis at single nucleotide resolution by selective 2'-hydroxyl acylation and primer extension (SHAPE). *J. Am. Chem. Soc.*, **127**, 4223–4231.

2. Lucks, J.B., Mortimer, S.A., Trapnell, C., Luo, S., Aviran, S., Schroth, G.P., Pachter, L., Doudna, J.A. and Arkin, A.P. (2011) Multiplexed RNA structure characterization with selective 2'-hydroxyl acylation analyzed by primer extension sequencing (SHAPE-Seq). *Proceedings of the National Academy of Sciences*, **108**, 11063–11068.

3. Mortimer, S.A., Trapnell, C., Aviran, S., Pachter, L. and Lucks, J.B. (2012) SHAPE-Seq: High-Throughput RNA Structure Analysis. *Curr Protoc Chem Biol*, **4**, 275–297.

4. Kladwang, W., VanLang, C.C., Cordero, P. and Das, R. (2011) Understanding the errors of SHAPE-directed RNA structure modeling. *Biochemistry*, **50**, 8049–8056.

5. Hajdin, C.E., Bellaousov, S., Huggins, W., Leonard, C.W., Mathews, D.H. and Weeks, K.M. (2013) Accurate SHAPE-directed RNA secondary structure modeling, including pseudoknots. *Proc. Natl. Acad. Sci. U.S.A.*, **110**, 5498–5503.

6. Reuter, J.S. and Mathews, D.H. (2010) RNAstructure: software for RNA secondary structure prediction and analysis. *BMC Bioinformatics*, **11**, 129.

7. Cordero, P., Lucks, J.B. and Das, R. (2012) An RNA Mapping DataBase for curating RNA structure mapping experiments. *Bioinformatics*, **28**, 3006–3008.
